# Tea plant (*Camellia sinensis*) lipid metabolism pathway modulated by tea field microbe (*Colletotrichum camelliae*) to promote disease

**DOI:** 10.1093/hr/uhad028

**Published:** 2023-02-21

**Authors:** Shouan Liu, Shuhan Zhang, Shengnan He, Xiaoyan Qiao, A Runa

**Affiliations:** Laboratory of Tea and Medicinal Plant Biology, College of Plant Sciences, Jilin University, Changchun 130062, China; Laboratory of Molecular Plant Pathology, College of Plant Sciences, Jilin University, Changchun 130062, China; Laboratory of Tea and Medicinal Plant Biology, College of Plant Sciences, Jilin University, Changchun 130062, China; Laboratory of Molecular Plant Pathology, College of Plant Sciences, Jilin University, Changchun 130062, China; Laboratory of Tea and Medicinal Plant Biology, College of Plant Sciences, Jilin University, Changchun 130062, China; Laboratory of Molecular Plant Pathology, College of Plant Sciences, Jilin University, Changchun 130062, China; Guangdong Provincial Key Laboratory of Tea Plant Resources Innovation and Utilization, Guangdong Academy of Agricultural Sciences Tea Research Institute, Guangzhou 510640, China; Laboratory of Tea and Medicinal Plant Biology, College of Plant Sciences, Jilin University, Changchun 130062, China; Laboratory of Molecular Plant Pathology, College of Plant Sciences, Jilin University, Changchun 130062, China

## Abstract

Tea is one of the most popular healthy and non-alcoholic beverages worldwide. Tea anthracnose is a disease in tea mature leaves and ultimately affects yield and quality. *Colletotrichum camelliae* is a dominant fungal pathogen in the tea field that infects tea plants in China. The pathogenic factors of fungus and the susceptible factors in the tea plant are not known. In this work, we performed molecular and genetic studies to observe a cerato-platanin protein CcCp1 from *C. camelliae*, which played a key role in fungal pathogenicity. △*CcCp1* mutants lost fungal virulence and reduced the ability to produce conidia. Transcriptome and metabolome were then performed and analysed in tea-susceptible and tea-resistant cultivars, Longjing 43 and Zhongcha 108, upon *C. camelliae* wild-type CCA and △*CcCp1* infection, respectively. The differentially expressed genes and the differentially accumulated metabolites in tea plants were clearly overrepresented such as linolenic acid and linoleic acid metabolism, glycerophospholipid metabolism, phenylalanine biosynthesis and metabolism, biosynthesis of flavonoid, flavone and flavonol etc. In particular, the accumulation of jasmonic acid was significantly increased in the susceptible cultivar Longjing 43 upon CCA infection, in the fungal CcCp1 protein dependent manner, suggesting the compound involved in regulating fungal infection. In addition, other metabolites in the glycerophospholipid and phenylalanine pathway were observed in the resistant cultivar Zhongcha 108 upon fungal treatment, suggesting their potential role in defense response. Taken together, this work indicated *C. camelliae* CcCp1 affected the tea plant lipid metabolism pathway to promote disease while the lost function of CcCp1 mutants altered the fungal virulence and plant response.

## Introduction

The beverage plant *Camellia sinensis* (L.) O. Kuntze originated in China and now has the benefits of massive job opportunities and wealth around the world [1]. Tea plant is a perennial woody plant and faces multiple biotic and abiotic stresses during its long life [1, 2]. Tea anthracnose is a disease that usually occurs on the mature tea leaves, damages the tea leaves, and influences tea yield and quality [3–6]. *Colletotrichum camelliae* is the dominant fungal pathogen in tea fields that infects tea plants [5, 6].

Generally, plants have evolved an immune system to defend against potential microbes [7, 8]. Plants respond to an infection through the following interconnected layers [9, 10]. One layer recognizes pathogen- (microbe-)associated molecular patterns (PAMPs or MAMPs) by pattern recognition transmembrane receptors (PRRs) and is called a PAMP- (or MAMP-)triggered immunity (PTI or MTI) [7–10]. The successful pathogens have evolved various virulence factors named as effectors to escape or suppress PTI/MTI in order to achieve successful infection [7, 8]. Thus, the other layer responds to microbe-specific effectors, which are recognized by NB-LRR proteins and are named effector-triggered immunity (ETI) [7–10]. In both layers, plants activate multiple downstream defense-signaling pathways (such as cell surface immune receptors, intracellular immune receptors, mitogen-activated protein kinases, transcription factors, hormones signaling, etc) and metabolic pathways (secondary metabolism) [11, 12].

Phytohormones regulate numerous aspects of plant growth and development and are involved in alleviating multiple biotic or abiotic stresses, by controlling either transcriptional or translational networks [11]. The jasmonic acid (JA), salicylic acid (SA), and ethylene (ET) are well known for regulating plant defense response against pathogens and are called the immunity hormones [11]. JA is biosynthesized from linolenic acid, the major fatty acid of plant cell membrane [2, 11]. Upon pathogen infection, the JA and ET signals are thought to operate synergistically as they induce similar defense genes [11]. Generally, JA/ET signaling played a role in plant defense to the necrotrophic pathogen, while SA signaling played a key role in plant resistance to the hemi-biotrophic and biotrophic pathogens [11]. It is now the case that the hormone pathways are intimately connected and that substantial crosstalk between pathways existed.

Next to phytohormones, secondary metabolites play important roles in plant defense [12, 13]. Plant metabolites with antimicrobial activity that are induced by various stresses are considered as molecular markers of disease resistance [13]. Previous works have found that tea and other plants contain high concentrations of anti-fungal components; some are phenolic compounds that provide the plant with a certain degree of basic resistance [6, 14, 19]. The eougakkicatecub-3-gallate, caffeine, and catechin were induced in tea plant after *Colletotrichum fructicola* infection, which could inhibit fungal mycelial growth [5, 15]. Other reports indicated certain flavonols have antimicrobial activity [16, 17]. For example, the cyanidin aglycones and quercetin were reported to inhibit *Colletotrichum gloeosporioides* conidial germination and hyphal growth [18, 19].

On the pathogen side, a few molecules are reported as MAMPs/PAMPs, such as chitin, chitosan, xylanase, glucans, etc [20–22]. Recent studies indicate proteins belonging to the cerato-platanin family have provided their MAMP/PAMP activity [23]. Cerato-platanin proteins (CPs) are a group of small, secreted, cysteine-rich proteins that have been implicated in biocontrol fungi, *Trichoderma* spp., that positively interact with plants [24]. In other reports, CPs are indicated in the virulence of certain plant pathogens including *Botrytis cinerea*, *Sclerotinia sclerotiorum*, etc [25–28]. However, there was no such information reported in tea field microbes, such as *C. camelliae.* In this work, we observed one cerato-platanin protein, named as CcCp1, played a key role in fungal pathogenicity based on genetic study. Further transcriptome and metabolome studies indicated CcCp1 protein modulated tea plant lipid metabolism pathway to promote disease development.

## Results

### CcCp1 encodes cerato-platanin protein

**Figure 1:**
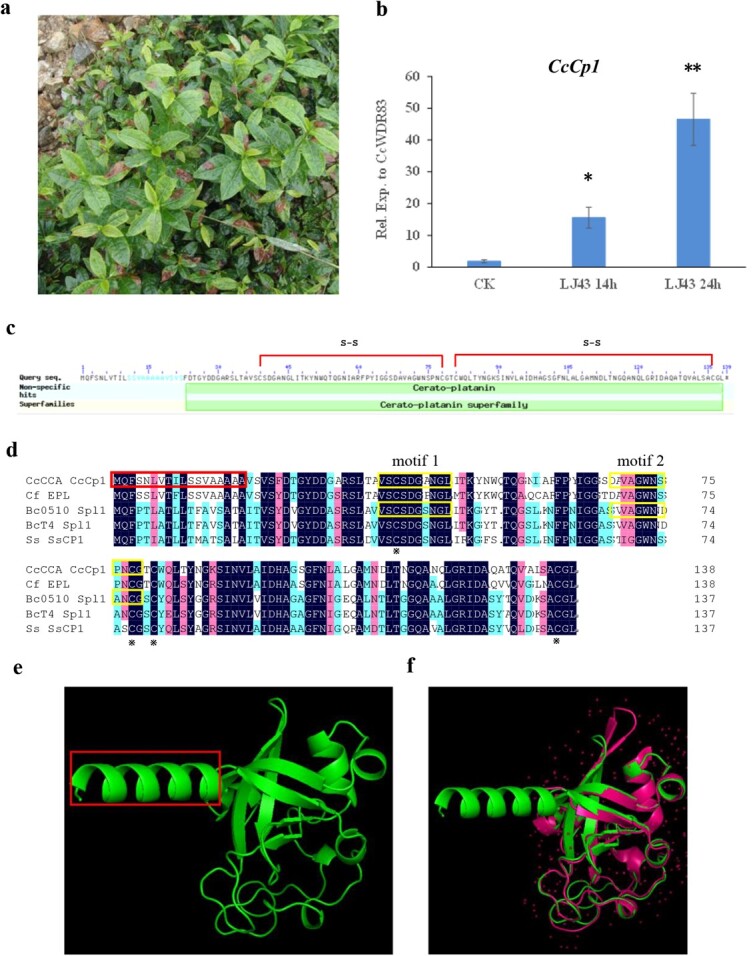
*Colletotrichum camelliae CcCp1* encodes cerato-platanin protein. **a** Phenotype of tea plant infected by *C. camelliae* in the field. **b** qPCR analysis of *CcCp1* in *C. camelliae* CCA upon interaction with tea cultivar Longjing 43. **c** Blast analysis indicates CcCp1 protein contained conserved domains of cerato-platanin protein. **d** Functional domains analysis of CcCp1. Alignment of CcCp1 protein with *Colletotrichum falcatum* EPL 349063 (XP_018151155.1), *Botrytis cinerea* B05.10 (XP_024545992), *B. cinerea* T4 (CCD43517), and *Sclerotinia sclerotiorum* 1980 UF-70 (XP_001585867.1). The red frame indicates the signal peptide. The yellow frame indicates the conserved motifs observed in *B. cinerea* B05.10 BcSpl1. **e** The predicted structure of CcCp1. The red frame indicates the signal peptide [30]. **f** The protein structure similarity of CcCp1 with known crystal structure of *Moniliophthora perniciosa* MpCP2 (the red structure) [30].

Tea cultivar Longjing 43 is quite susceptible to *C. camelliae* in the field and expression of many genes are induced in the fungi during disease development. We next want to know which pathogenic mechanism is involved (Fig. 1a). CcCp1 was analysed by searching the *C. camelliae* genome sequence using BlastP. The full length of *CcCp1* is 417 base pairs encoding a protein containing 138 amino acid residues with predicted N-terminal signal peptide (1–18 AA), suggesting that it may be a secreted protein (Fig. 1c; Fig. S1, see online supplementary material). Structurally, the putative ‘cerato-platanin (CP, Pfam: PF07249)’ domain contains four well-conserved cysteine residues which might form two disulfide bonds (19–138 AA, Fig. 1c and d). CP proteins occur in the fungal cell walls and are involved in the host–plane interaction and they induce both cell necrosis and phytoalexin synthesis [24–29].

Amino acid sequence of CcCp1 was then aligned with cerato-platanin proteins identified in *Colletotrichum* spp. and several other ascomycetes species (Fig. 1d). The sequences of CP proteins from *Colletotrichum* spp. were also used to build a neighbor-joining tree (Fig. S2, see online supplementary material). CcCp1 was clustered together and had higher similarity with *Colletotrichum* spp. In addition, CcCp1 showed the higher identity percentage with the CP proteins CfEPL of fungal *Colletotrichum falcatum* EPL (86%) [29], BcSpl1 of fungal *B. cinerea* (61% of B05.10 and 59% of T4) [ 25], and SsCP1 of fungal *S. sclerotiorum* (58%) [28] (Fig. 1d). The protein structure of CcCp1 was then predicted according to I-TASSER [30]. The predicted 3-dimensional (3D) structure showed that CcCp1 contained two α-helices and six β-strands, which were highly similar to *Moniliophthora perniciosa* MpCP2 (Fig. 1e and f) with one more α-helice at the N-terminal site [31]. Sequence alignment analysis with *B. cinerea* strains B05.10 and T4 indicated that CcCp1 may have two conserved motifs cooperate to induce necrosis, and the peptide CSDGXNGL in the first motif was highly conserved in CPs (Fig. 1d) [ 27].

To investigate whether cerato-platanin was involved in the interaction between *C. camelliae* and tea plant, the expression profile of *CcCp1* was analysed by qRT-PCR. As indicated in Fig. 1b, expression of *CcCp1* was induced during *C. camelliae* infection tea plant cultivar Longjing 43. At 14 hours, expression level was about 16-fold higher in *C. camelliae* than CK while it increased to 48-fold at 24 hours. This result indicated *CcCp1* was induced in *C. camelliae* during infection, which could play a role in tea plant–fungus interaction.

### CcCp1 plays an important role in conidia production and fungal virulence

**Figure 2:**
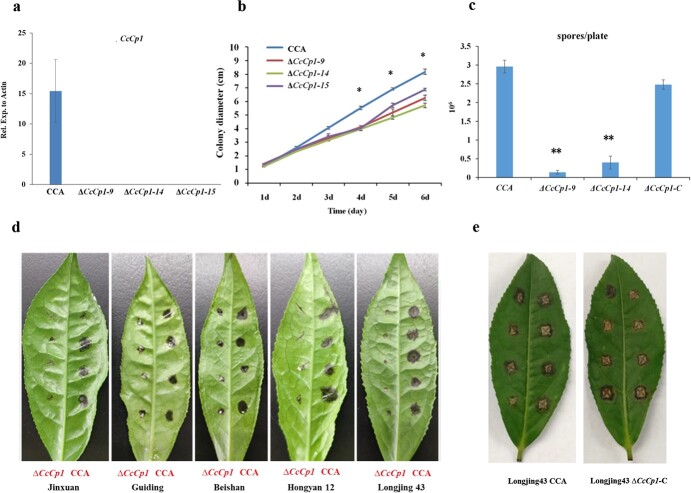
*Colletotrichum camelliae* CcCp1 involves in fungal growth, pathogenicity, and conidia production. **a** qPCR analysis of *CcCp1* gene expression in *C. camelliae* CCA and △*CcCp1* mutants. **b** Growth difference of *C. camelliae* CCA and △*CcCp1* mutants. **c** Conidia productivity of *C. camelliae* CCA, △*CcCp1–9*, △*CcCp1–14,* and △*CcCp1*-C strains. Y-axis indicated mean conidia concentration of indicated strains in CM plates. **d** Infection phenotypes of *C.camelliae* CCA (right side) and △*CcCp1* (left side) on leaves of indicated tea cultivars (5 dpi). These include Jinxuan, Guiding, Beishan, Hongyan12, and Longjing 43. **e** Phenotype of tea plant Longjing 43 infection with *C. camelliae* CCA and Δ*CcCp1*-C complement line. The Δ*CcCp1*-C complement line restores wild-type CCA virulence.

To reveal the molecular role of *CcCp1* in *C. camelliae*, *CcCp1* mutants (∆*CcCp1*) were generated by the gene replacement method (Fig. S3a, see online supplementary material). ∆*CcCp1* was obtained by single conidium isolation with homozygotic resistance and further verified by PCR and qPCR (Fig. 2a). The complemented strains (∆*CcCp1-*C) were obtained by introducing *CcCp1* into ∆*CcCp1* mutant with neomycin resistance and examined by PCR (Fig. S3b and c, see online supplementary material).

To explore the possible role of *CcCp1* in *C. camelliae*, fungal growth rate and conidiation ability were compared. The growth speed of aerial hyphae in *C. camelliae* wild-type CCA was a bit faster than ∆*CcCp1* mutants (∆*CcCp1–9*, ∆*CcCp1–14*, and ∆*CcCp1–15*), suggesting CcCp1 contributed to fungal growth (Fig. 2b). Surprisingly, the ability to produce conidia was remarkably different among these tested strains. The conidia production was significantly reduced in ∆*CcCp1* mutants compared with that of the wild-type CCA (Fig. 2c). ∆*CcCp1**-***C could restore the conidiation yield of ∆*CcCp1* mutants (Fig. 2c). These data indicated that *CcCp1* was involved in the regulation of *C. camelliae* growth and conidiation.

To know if *CcCp1* contributed to *C. camelliae* virulence, we tested the virulence of ∆*CcCp1* on tea plants by detached leaf inoculation method. The disease intensity was determined by examining lesion size at 5 days after inoculation with equal amounts of conidia from *C. camelliae* CCA and ∆*CcCp1* (Fig. 2d). Tea plant different cultivars were used. These included, Jinxuan, Guiding, Beishan, Hongyan 12, and Longjing 43. Under experimental conditions, the typical anthracnose symptoms, e.g. necrotic lesions, were observed in tea leaves inoculated with *C. camelliae* CCA (Fig. 2d). At 5 days post-inoculation, the lesion size caused by ∆*CcCp1* was much smaller compared with the wild-type CCA (Fig. 2d). ∆*CcCp1-*C complement strain could restore the virulence of CCA (Fig. 2e). These data indicated that the loss function of *CcCp1* resulted in reducing fungal virulence, revealing the contribution of *C. camelliae CcCp1* for pathogenicity to tea plants.

### Transcriptome analysis indicates differentially expressed genes changed in resistant and susceptible tea cultivars towards *C. camelliae* CCA and Δ*CcCp1* infection

Because tea plant Longjing 43 was susceptible to the wild-type strain CCA while Δ*CcCp1* changed fungal virulence, we next wanted to know which genes were affected in tea plant response to *C. camelliae* infection. Previous studies revealed the resistance levels toward *C. camelliae* CCA were very different among tea cultivars; while tea cultivar Longjing 43 was more susceptible, the cultivar Zhongcha 108 was more resistant [4]. Zhongcha 108 was the mutation line of Longjing 43 and both cultivars are grown widely in China. Thus, tea cultivars Longjing 43 and Zhongcha 108 were used for transcriptomic analysis. We harvested *C. camelliae* wild-type CCA (CC) and Δ*CcCp1* mutants (Cpm) spray-inoculated tea leaves from tea cultivar Longjing 43 (LJ) and Zhongcha 108 (ZC) at a time point 24 hours post infection (hpi) for RNA sequencing (CC 24 h and Cpm 24 h). Mock-treated tea leaves were used as control (CK). About 791 million high quality reads in total and around 6.6 G base pairs from a single library on average were obtained. Table S1 (see online supplementary material) indicates an overview of the RNA sequence reads for all libraries. A total of 772 million validated high-quality reads were finally generated (97.5%). The reads were then mapped to the tea plant genome. Transcripts were further normalized by counting Fragments Per Kilobase Million (FPKM) and the differentially expressed genes (DEGs) were defined by considering the fold change and the false discovery rated [16].

To identify genes in tea plant response to *C. camelliae*, we compared and analysed the statistically significantly differentially changed genes (altered $\ge$ two-folds, P ≤ 0.05; SSTF) between *C. camelliae* CCA-treated (CCLJ, CCZC) or *C. camelliae* Δ*CcCp1*-treated (CPmLJ, CPmZC) with un-treated tea cultivars (CKLJ, CKZC), respectively.

**Figure 3:**
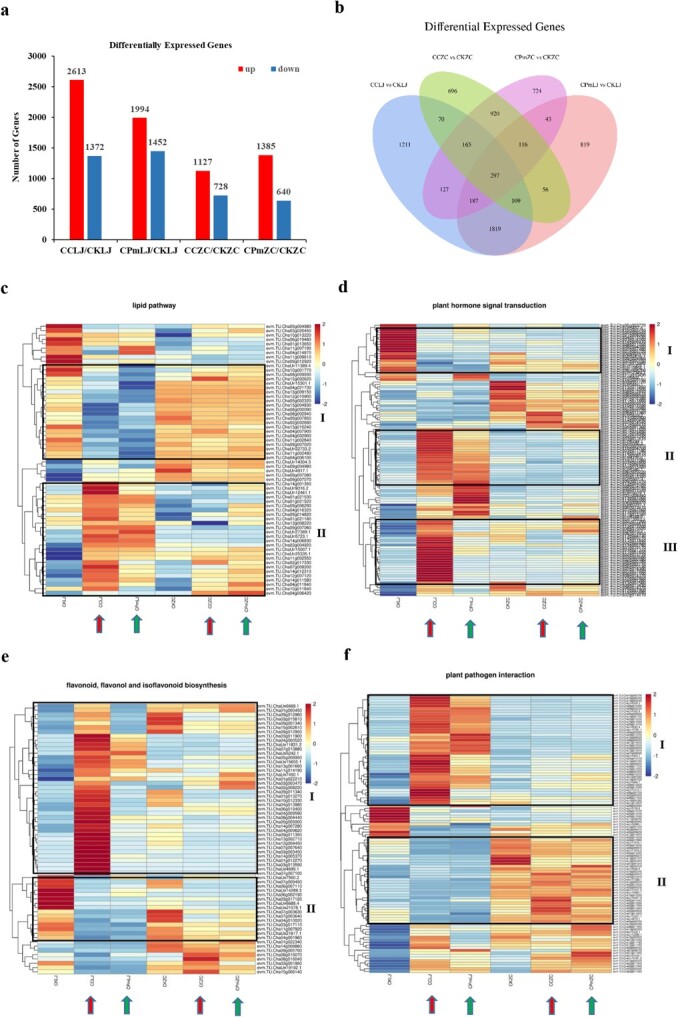
Transcriptomic analysis revealed differentially expressed genes in tea plant upon *C. camellia* CCA and △*CcCp1* infection. **a** Numbers of differentially expressed genes (≥2-fold; P ≤ 0.05) in tea plant Longjing 43 (LJ) and Zhongcha 108 (ZC) upon *C. camellia* CCA (CC) or △*CcCp1* (CPm) infection at 24 h or without infection (CK) by RNA-seq. **b** Venue analysis of the overlap genes between CCA affected and Δ*CcCp1* affected differentially regulated genes. (**c**–**f**). Heatmap analysis of differentially expressed genes in tea plants upon *C. camellia* CCA and △*CcCp1* infection. The arrows with red color indicated CCA infected tea plants while the green arrows indicated △*CcCp1* infected tea plants. **c** The heatmap analysis of DEGs in lipid pathway. The framed indicated genes increased or decreased in CCA-infected Longjing 43 compared with CKLJ while their expression levels were altered in △*CcCp1*-infected Longjing 43 or both fungi affected Zhongcha108. **d** The heatmap analysis of DEGs involved in plant hormone signal transduction. **e** The heatmap analysis of DEGs involved in flavonoid, flavonol, and isoflavonoid biosynthesis. **f** The heatmap analysis of DEGs involved in plant pathogen interaction. All frames indicate differentially expressed genes upon CCA and △*CcCp1* treatments.

During *C. camelliae* CCA-tea cultivar Longjing 43 compatible interaction for 24 hours, around 3985 SSTF genes were obtained in CCA-treated tea plant (CCLJ) compared with non-infected control (CKLJ), with 2613 genes being up-regulated and 1372 genes being down-regulated (Fig. 3a). When we compared the Δ*CcCp1*-treated plant (CPmLJ) with CKLJ, around 3446 SSTF genes were identified with 1994 genes up-regulated and 1452 genes down-regulated (Fig. 3a). These data indicate both CCA and Δ*CcCp1* affected tea plant Longjing 43 transcript changes and we are interested in which genes are involved. The overlap genes between CCA- and Δ*CcCp1-*affected tea plant Longjing 43 were about 2412, while 1573 genes were observed only in CCA-infected Longjing 43 and 1034 genes were observed only in Δ*CcCp1-*infected (Fig. 3b).

During the incompatible interaction between *C. camelliae* CCA-tea cultivar Zhongcha 108, around 1855 SSTF genes were obtained in CCA-treated plant (CCZC) compared with non-infected control (CKZC), with 1127 genes being up-regulated and 728 genes being down-regulated (Fig. 3a). During *C. camelliae* Δ*CcCp1*-Zhongcha 108 interaction, around 2025 SSTF genes were identified with 1385 genes up-regulated and 640 genes down-regulated (Fig. 3a). These data indicated CCA and Δ*CcCp1* also affected tea plant Zhongcha 108 transcripts changes but the total numbers of SSTFs were lower than that on Longjing 43. The overlap genes between CCA- and Δ*CcCp1*-affected tea plant Zhongcha 108 were about 1154, while 931 genes were observed only in CCA-infected tea plant Zhongcha 108 and 1081 genes were observed only in Δ*CcCp1*-infected (Fig. 3b).

### *C. camelliae* CcCp1-altered transcription changes associated with tea plant defense responses between cultivars Longjing 43 and Zhongcha 108

Because *C. camelliae* wild-type CCA and Δ*CcCp1* infection triggered tea plant transcriptional reprogramming, we next wanted to know which pathways were involved. KEGG pathway and GO term analysis were performed, respectively. Based on KEGG analysis, the overlap SSTF genes between CCA and Δ*CcCp1* infected Longjing 43 were clearly enriched in several common pathways including α-linolenic acid metabolism, fatty acid degradation, phenylpropanoid biosynthesis, flavonoid biosynthesis, isoflavonoid biosynthesis, flavone and flavonol biosynthesis, and zeatin biosynthesis (Fig. S4a, see online supplementary material). Based on GO terms, the overrepresented categories included flavonoid biosynthetic process, flavonoid glucuronidation, quercetin-O-glucosytransferase activity, protein kinase activity, etc (Fig. S4b, see online supplementary material).

KEGG and GO analysis of the overlap genes between CCA and Δ*CcCp1* infected Zhongcha 108 revealed similar enrichments such as flavonoid biosynthesis, isoflavonoid biosynthesis, phenylpropanoid biosynthesis, fatty acid elongation, plant hormones signal transduction, etc (Fig. S5a and b, see online supplementary material). As a difference, the ABC transporter, MAPK signal pathway, cutin biosynthetic process, brassinosteroids (biosynthesis, homeostasis, and metabolism) were enriched in the overlap genes between CCA and Δ*CcCp1*-infected Zhongcha 108 (Fig. S5a and b, see online supplementary material). As the genes involved in these categories/pathways are associated with plant defense response, here GO and KEGG results indicate the SSTF genes play roles in tea plant–*C. camelliae* interaction.

For example, many genes involved in lipid pathways were increased in CCA-infected Longjing 43 than Δ*CcCp1*-treated (Fig. 3c, frame II). These include: six genes encoding 3-ketoacyl-CoA synthase, three genes encoding ketoacyl-ACP synthase, two genes encoding acyl ACP thioesterase, and one gene encoding 3-hydroxyacyl-ACP dehydratase, long chain acyl-CoA synthetase, short-chain dehydrogenase or phospholipase, lipoxygenase, allene oxide cyclase, respectively (Additional file S1, see online supplementary material). These genes are involved in lipid synthesis, fatty acid biosynthesis, fatty acid catabolism, phospholipid catabolism, etc. However, many of them are not highly increased in CCA-infected Zhongcha 108 compared with Δ*CcCp1*, suggesting CcCp1 played a special role in activating tea plant lipid pathway in Longjing 43, more than Zhongcha 108 (Fig. 3c, frame II; Additional file S1, see online supplementary material). In addition, expression of many other genes was decreased and clustered together in both CCA and Δ*CcCp1*-infected Longjing 43 compared with CKLJ (Fig. 3c, frame I). However, expression of these genes in tea plant Zhongcha108 was not affected upon fungal infection and kept in a higher expression level, suggesting a different role of lipids existed between Longjing 43 and Zhongcha 108 (Fig. 3c, frame I; Additional file S1, see online supplementary material).

Next, SSTF genes involved in plant hormone transduction pathways were also overrepresented including abscisic acid receptor-like protein, DELLA protein, auxin response factor, ethylene response factor, ethylene receptor like, auxin transporter, auxin-induced and responsive protein, etc (Fig. 3d; Additional file S2, see online supplementary material). In particular, 13 genes encoding auxin response and transduction, seven genes encoding bHLHs, four genes encoding serine/threonine-protein kinase, the genes encoding DELLA and other proteins are highly expressed in CCA-infected Longjing 43 than Δ*CcCp1*. (Fig. 3d, frames II, III; Additional file S2, see online supplementary material)*.* Interestingly, the expression levels of these genes were also lower in Zhongcha108, suggesting a different role. We further observed several genes clustered together especially in certain conditions, such as CKLJ (Fig. 3d, frame I), CKZC, CPmLJ, or CCZC (Fig. 3d; Additional file S2, see online supplementary material)*.*

In addition, many genes involved in flavonoid, flavonol, and isoflavonoid biosynthesis pathways were highly increased in CCA-attacked Longjing 43 compared with Δ*CcCp1* (Fig. 3e, frame I; Additional file S3, see online supplementary material)*.* These include genes encoding acetyltransferase, anthocyanidin synthase, BAHD acyltransferase, chalcone isomerase, chalcone synthase, cinnamic acid 4-hydroxylase, leucoanthocyanidin reductase, dihydroflavonol 4-reductase, Shikimate O-hydroxycinnamoyltransferase, flavanone 3-hydroxylase, flavonol synthase, flavonoid 3′,5′-hydroxylase, UDP-glycosyltransferase, etc (Additional file S3, see online supplementary material). Similarly, many of these genes were not highly expressed in Zhongcha 108 upon fungal infection.

Additionally, we observed the genes enriched in plant–pathogen interaction. These include: disease resistance protein, calcium-dependent protein kinase, LRR receptor-like serine/threonine-protein kinase, calcium-binding protein, ethylene-responsive transcription factor, WRKY transcription factor, etc (Fig. 3f; Additional file S4, see online supplementary material). Several clusters of differentially expressed genes were clustered. In one cluster, expression of genes was increased in CCA and Δ*CcCp1-* infected Longjing 43 compared with CKLJ, while the induction of these genes was not observed in Zhongcha 108 (Fig. 3f, frame I). In another cluster, expression of genes was higher in Zhongcha 108 compared with Longjing 43 (Fig. 3f, frame II). Induction and repression of genes expression during tea plant different cultivars in *C. camelliae* interaction, suggesting different mechanisms might be involved.

### Metabolomics analysis reveals differentially accumulated metabolites (DAMs) in tea cultivars upon *C. camelliae* CCA and Δ*CcCp1* infection

To explore the change of metabolites between tea cultivars, Longjing 43 and Zhongcha 108, upon *C. camelliae* wild-type CCA and Δ*CcCp1* infection, a metabolome approach was performed and analysed as in [16] (*n* = 36; Fig. S6 and S7. see online supplementary material). Final statistics revealed that 10 597 metabolites were annotated in tea plant Longjing 43 while 23 937 metabolites were annotated in Zhongcha 108 (Table S2, see online supplementary material).

**Figure 4:**
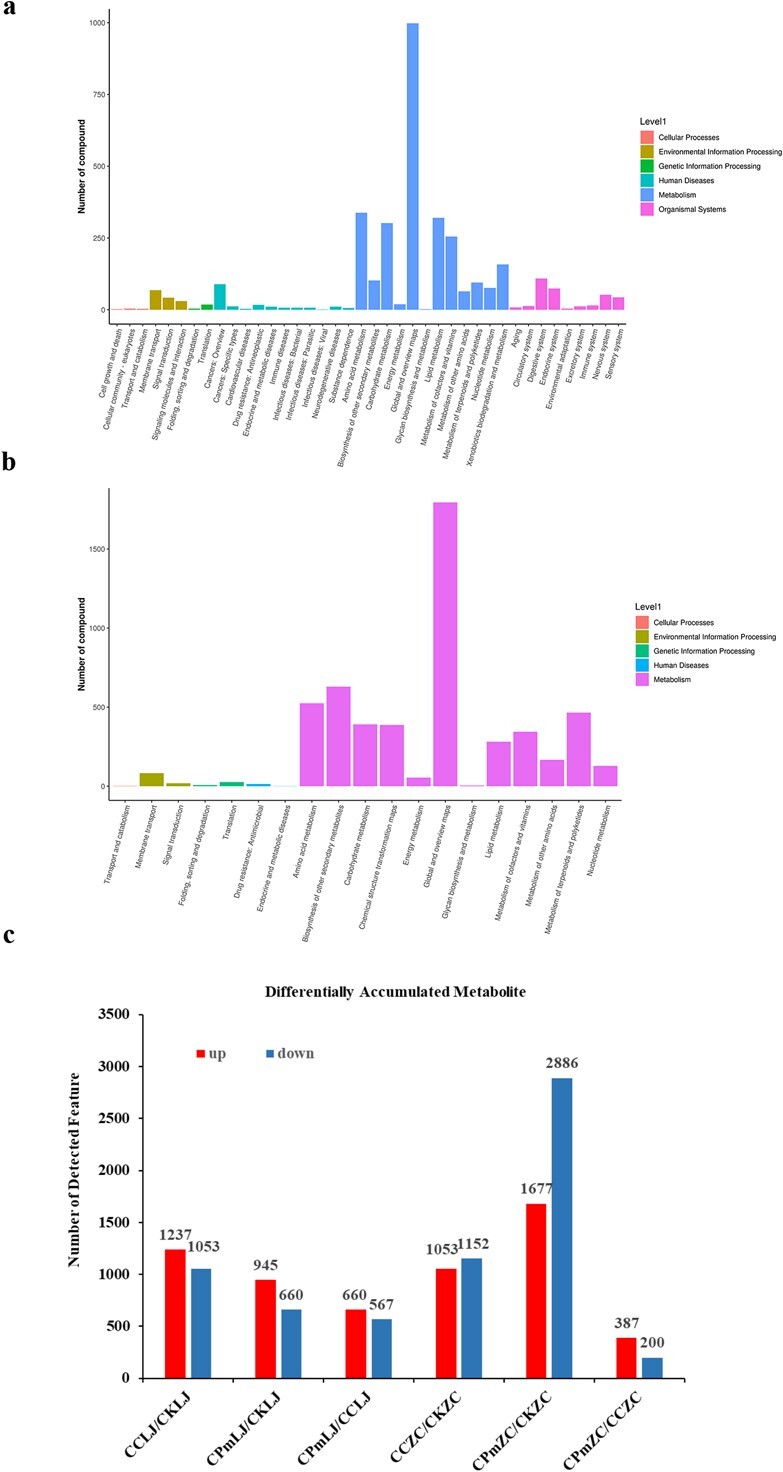
Identification of the metabolites observed in tea cultivars upon *C. camelliae* CCA and △*CcCp1* treatments or un-treatment. The metabolites observed in Longjing 43 (**a**) and Zhongcha 108 (**b**) assigned to the KEGG pathway classification. The x-axis represents level-1 terms of the KEGG pathway and the y-axis represents the number of metabolites identified. **c** Numbers of differentially accumulated metabolites (DAMs) in tea cultivars (Longjing 43: LJ, Zhongcha 108: ZC) upon *C. camellia* CCA (CC) or △*CcCp1* (CPm) treated at 24 h or without treatment (CK).

Next, the identified metabolites were assigned to the HMDB and KEGG databases, respectively [16]. About 9160 metabolites were enriched in 25 HMDB super class in Longjing 43 (Table S2; Fig. S8a, see online supplementary material), while around 20 580 metabolites were enriched in 27 HMDB super class in Zhongcha 108 (Table S2; Fig. S8b, see online supplementary material). Interestingly, about 49.4% features (44 311 out of 89 657) were enriched in lipids and lipid-like molecules super-class (Fig. S8a, framed, see online supplementary material), suggesting these compounds played a role in Longjing 43–*C. camelliae* interaction. Similar results were observed in Zhongcha 108, in which about 46.5% features associated with lipids and lipid-like molecules were enriched (Fig. S8b, framed, see online supplementary material). For the KEGG analysis, 6 second-grade categories were observed in Longjing 43 (Fig. 4a), while 5 second-grade categories were observed in Zhongcha 108 (Fig. 4b). The compounds observed in the study are mostly classed in ‘metabolism’ (Fig. 4a and b). We further analysed the KEGG pathway (KEGG level 3) of all candidate metabolisms. Several stress-related metabolism pathways are particularly enriched (Figs S9 and S10, see online supplementary material).

To provide a deep overview of the metabolic changes among *C. camelliae* CCA-infected Longjing 43 and Zhongcha 108, *C. camelliae* Δ*CcCp1*-infected Longjing 43 and Zhongcha 108, and the un-infected control plants (CKLJ, CKZC), several quality control (QC) parameters for the quantification were performed as previously [16]. These include principal component (PC), coefficient of variation (CV), and normalized intensity. The PC analysis showed separations between treatments (Fig. S11, see online supplementary material). As a result, 12 690 and 33 729 high-quality features were used for differential analysis (Table S2, see online supplementary material). An overview of the metabolite profiling of tea plant CK and *C. camelliae* CCA or Δ*CcCp1* treated tea plants were shown in Fig. S12 (see online supplementary material), the variations in the metabolomes between CK and CCA or Δ*CcCp1* were observed.

According to the quantitative results of the identified metabolites, the DAMs between different comparisons were analysed (Fig. 4c; Table S3; Fig. S13, see online supplementary material). The statistical analysis identified 2290 significant DAMs in the comparison between CCA-infected Longjing 43 (CCLJ) and control (CKLJ), with a total of 1237 and 1053 metabolites presented as being up-regulated and down-regulated, respectively. When compared between Δ*CcCp1*-infected Longjing 43 (CPmLJ) and control (CKLJ), 1605 significant DAMs were observed, with 945 being up-regulated and 660 being down-regulated. The significant DAMs between CCLJ and CPmLJ were about 1227, with 660 being up-regulated and 567 being down-regulated.

In Zhongcha 108, when compared with untreated control, the DAMs upon *C. camelliae* CCA and △*CcCp1* treatments were about 2205 and 4563, respectively (Fig. 4c; Table S3, see online supplementary material). Interestingly, about 63% of DAMs were down-regulated in Δ*CcCp1*-infected Zhongcha 108 (CPmZC) compared with control (CKZC) (Fig. 4c; Table S3, see online supplementary material). The DAMs between CCZC and CPmZC were about 587, with 387 being up-regulated and 200 being down-regulated (Fig. 4c; Table S3, see online supplementary material).

All DAMs were next assigned to diverse metabolic categories (Figs S14 and S15; Additional file S5, see online supplementary material). In Longjing 43, these pathways include biosynthesis of unsaturated fatty acids, linolenic acid metabolism, linolenic acid metabolism, phenylalanine metabolism, phenylalanine, tyrosine, and tryptophan biosynthesis, etc. Particularly, the metabolites in lipid metabolism such as linoleate, linolenic Acid, 13-OxoODE, 9,12,13-TriHOME, traumatin, and jasmonic acid are observed (Additional file S5, see online supplementary material). In Zhongcha 108, similar pathways are observed including the metabolites in linoleic acid and linolenic acid metabolism, glycerophospholipid metabolism, flavonoid biosynthesis, phenylpropanoid biosynthesis and metabolism, etc. In particular, the jasmonic acid, alpha-dimorphecolic acid, 9,10-Dihydroxy-12Z-octadecenoic acid, lysophosphatidylcholine, lysophosphatidylethanolamine, quercetin, 4-coumarate, trans-2-hydroxycinnamate, and coumarin are observed (Additional file S5, see online supplementary material). Our previous work had shown that polyunsaturated fatty acids, such as linolenic acid and linolenic acid could be catalyzed into hydroperoxides by tea plant lipoxygenases (LOXs) pathway [2]. Subsequently, they are catalyzed to various metabolites including 9(S)-HODE, 13-OxoODE, 9,12,13-TriHOME, 9,10-DiHOME, 12-OPDA, and jasmonic acid. The accumulation of metabolism in lipid metabolism pathways indicated their potential role in tea plant–*C. camelliae* interaction.

### Integrated transcriptome and metabolomic analysis indicating CcCp1 involved in fungal promoting disease development

**Figure 5:**
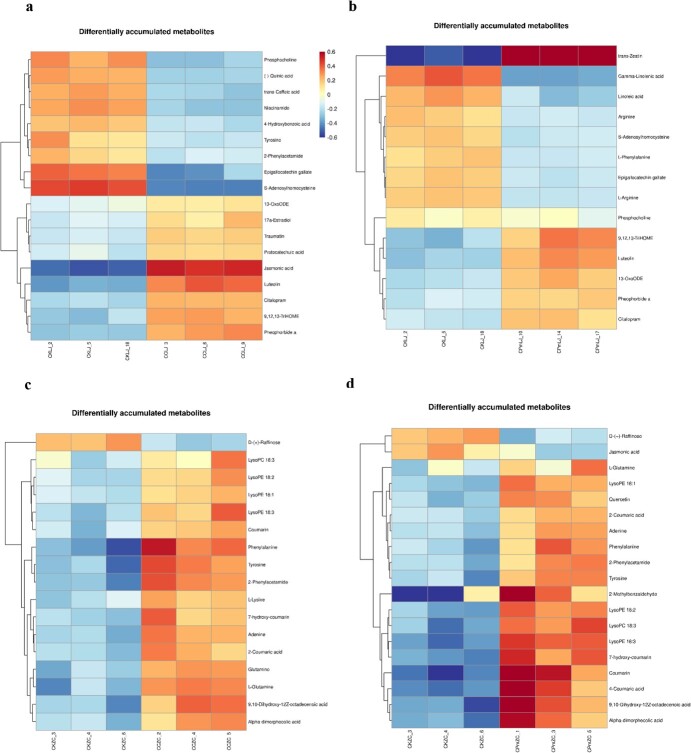
Heatmap analysis of differentially accumulated metabolites involved in tea cultivars upon *C. camelliae* CCA and △*CcCp1* treatments compared with CK. **a** Heatmap analysis of DAMs involved in tea cultivar Longjing 43 upon *C. camelliae* CCA infection compared with CKLJ. **b** Heatmap analysis of DAMs involved in tea cultivar Longjing 43 upon *C. camelliae* △*CcCp1* infection compared with CKLJ. **c** Heatmap analysis of DAMs involved in tea cultivar Zhongcha 108 upon *C. camelliae* CCA infection compared with CKZC. **d** Heatmap analysis of DAMs involved in tea cultivar Zhongcha 108 upon *C. camelliae* △*CcCp1* infection compared with CKZC.

To reveal the association between genes and metabolites in similar biological processes, a comprehensive analysis of transcriptome and metabolome was performed using Pearson’s Correlation Coefficient as previously [16]. When comparing the *C. camelliae* CCA-treated Longjing 43 with CKLJ, our results indicated 1420 SSTF genes participated in 136 pathways, while 18 DAMs were involved in 13 pathways (Fig. 5a; Additional file S6, see online supplementary material). When comparing the *C. camelliae* Δ*CcCp1*-treated Longjing 43 with CKLJ, our results indicated 1192 SSTF genes participated in 132 KEGG pathways, while 14 DAMs were involved in 11 KEGG pathways (Fig. 5b; Additional file S7, see online supplementary material).

During Zhongcha 108 interaction with *C. camelliae* CCA, 856 SSTF genes participating in 119 pathways were observed, while 17 DAMs involved in 25 pathways were presented (Fig. 5c; Additional file S6, see online supplementary material). In addition, 886 SSTF genes participated in 128 KEGG pathways, while 19 DAMs involved in 28 KEGG pathways were observed in Δ*CcCp1*-treated Zhongcha 108 compared with control (Fig. 5d; Additional file S7, see online supplementary material).

**Figure 6:**
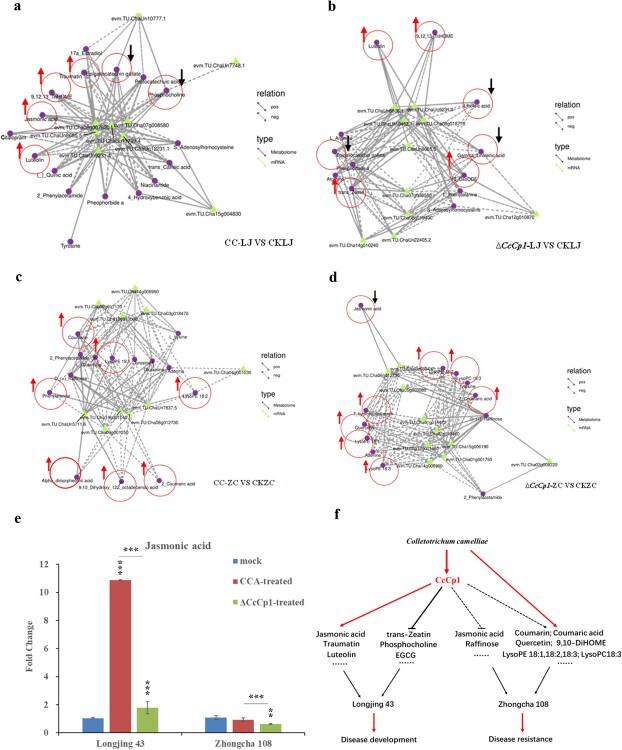
The putative genetic and metabolic regulatory networks during tea cultivars–*C. camelliae* interaction and the related working model. **a**–**d** The networks were based on identified differentially accumulated metabolites (DAMs) and key genes in the indicated treatments (Pearson correlation coefficients ≥0.9, *P* < 0.05). The arrows indicate the comparison of metabolite changes between treatment and control (red arrow: increased; black arrow: decreased). **a** The putative regulatory networks between *C. camelliae* CCA-treated and un-treated Longjing 43. **b** The putative genetic and metabolic regulatory networks between *C. camelliae* Δ*CcCp1*-treated and un-treated Longjing 43. **c** The putative genetic and metabolic regulatory networks between *C. camelliae* CCA-treated and un-treated Zhongcha 108. **d** The putative genetic and metabolic regulatory networks between *C. camelliae* Δ*CcCp1*-treated and un-treated Zhongcha 108. **e** Fold changes of jasmonic acid in tea cultivar Longjing 43 and Zhongcha 108 upon *C. camelliae* CCA and Δ*CcCp1* treatments or untreated control plants (mock), respectively. The significance was carried out between treatment and each control, between treatments in Longjing 43 or Zhongcha 108, respectively. (^**^P ≤ 0.01, ^***^P ≤ 0.001). Take untreated tea plants Longjing 43 and Zhongcha 108 as 1.0, respectively. **f** The role of CcCp1 in tea plant–*C. camelliae* interaction. Model indicates the key role of CcCp1 in *C. camelliae* suppressing tea plant defense and promoting disease development. Upon interaction with susceptible tea plant cultivar Longjing 43, *C. camelliae* CcCp1 is increased, which would affect plant lipid pathway and induce plant metabolites biosynthesis such as jasmonic acid, traumatin, and luteolin, while suppressing the biosynthesis of EGCG, phosphocholine, and trans-zeatin, and thus causing plant disease. In the resistant tea cultivar Zhongcha 108, *C. camelliae* CcCp1 seems not to induce jasmonic acid accumulation. *C. camelliae* infection enhances the induction of compounds in glycerophospholipid metabolism and phenylalanine metabolism pathway, which might have a defense response.

The comprehensive analysis between metabolome and transcriptome observed in Longjing 43 and Zhongcha 108 is shown (Fig. 6a–d; Additional files S6 and S7, see online supplementary material). Specially, the metabolism associated with lipid metabolism is both observed in CCA- and Δ*CcCp1*-infected tea plant cultivars indicating their potential role in CcCp1-mediated tea plant disease development or defense responses (Fig. 6a–d; Tables S4 and S5; Additional files S6 and S7, see online supplementary material).

In linoleic acid metabolism pathway, the accumulation of 9,12,13-TriHOME and 13-OxoODE were increased in both CCA- and Δ*CcCp1*-infected tea plant Longjing 43 compared with control (CKLJ) (Fig. 6a and b), while the accumulation of linoleic acid was decreased in Δ*CcCp1*-infected Longjing 43 compared with CKLJ or CCLJ (Fig. 6b; Fig. S16, see online supplementary material). In the linolenic acid metabolism pathway, the accumulation of traumatin and jasmonic acid were increased in CCA-treated Longjing 43 compared with CKLJ (Fig. 6a, e, and f), while the accumulation of gamma-linolenic acid decreased in Δ*CcCp1*-infected tea plant compared with CKLJ or CCLJ (Fig. 6b; Fig. S16, see online supplementary material).

Intriguingly, the accumulation of linoleic acid, gamma-linolenic acid, and jasmonic acid were all dramatically decreased in Δ*CcCp1*-treated Longjing 43 compared with CCA-treated (Fig. S16, see online supplementary material; Fig. 6e). Our results revealed *C. camelliae* CcCp1 affected unsaturated fatty acids metabolism in tea plant Longjing 43. Pathogen infection or PAMPs treatment can both trigger plant accumulation of jasmonic acid or enhance the expression of the jasmonic acid biosynthetic pathway genes. In citrus, transcriptomic and metabolomic analyses of the wax-deficient mutant showed jasmonic acid-mediated defense towards fungal pathogen [32]. However, here the accumulation of jasmonic acid was dramatically induced in CCA-infected Longjing 43 (about 10.9-fold more than CK) while the induction decreased upon Δ*CcCp1* infection (about 1.8-fold more than CK) (Fig. 6e). If considering that Longjing 43 is susceptible towards *C. camelliae* CCA, here the high induction of jasmonic acid in Longjing 43 by CCA may not play a role in plant defense, otherwise involved in plant disease development under the fungal CcCp1 dependent manner (Fig. 6f).

In addition, phosphocholine is the phosphate of choline and belongs to the choline phospholipids. It is a conjugate acid of choline phosphate. The accumulation of phosphocholine was more decreased in CCA-treated Longjing 43 than CK while it was partly recovered in Δ*CcCp1*-infected tea plant, suggesting the compound was also involved in Longjing 43–CCA interaction (Figs 5a and b and 6a, b and f). Taken together, *C. camelliae* CcCp1 might target the plant cell membrane to modulate the metabolism of linoleic acid, linolenic acid, or choline phospholipids in Longjing 43 during infection.

Similar to the results of Longjing 43, the accumulation of metabolites in linoleic acid, linolenic acid, and glycerophospholipid metabolism pathways were observed during Zhongcha 108–*C. camelliae* interaction (Fig. 6c and d; Table S5; Additional files S6 and S7, see online supplementary material). Different to the results of Longjing 43, here the compounds of LysoPC18:3, LysoPE18:1, LysoPE 18:2, LysoPE 18:3, alpha-dimorphecolic acid (9S-HODE), 9,10-Dihydroxy-12Z-octadecenoic acid (9,10-DiHOME), and jasmonic acid were observed in Zhongcha 108 upon both *C. camelliae* CCA and Δ*CcCp1* treatments. Most of the compounds were significantly induced in Zhongcha 108, except jasmonic acid (Fig. 6e and f). If we considered the resistance of Zhongcha 108 towards *C. camelliae*, these highly accumulated compounds might contribute to plant defense because the lipids and associated fatty acids played crucial roles in plant immunity.

Except plant lipid pathway, the compounds such as epigallocatechin gallate (EGCG), coumarin, 7-hydroxy-coumarin, 2-coumaric acid, 4-coumaric acid, luteolin, and quercetin are observed. Flavonoids are polyphenolic compounds ubiquitous in plants which contribute to plant stress adaptation, fruit growth, and human healthy [33]. These compounds are observed in various plant various tissues at different stages, and many have an anti-microbe function [14, 19, 34]. The EGCG was significantly decreased in CCA-infected tea plant Longjing 43 compared with control; however, biosynthesis of the compound was partly recovered in Δ*CcCp1*-infected Longjing 43 (Fig. 6a and b; Fig. S16, see online supplementary material). Interestingly, in the resistant tea cultivar Zhongcha 108, the accumulation of coumarin, 7-hydroxy-coumarin, 2-coumaric acid, 4-coumaric acid, luteolin, and quercetin were increased upon both CCA and Δ*CcCp1* infection (Fig. 6c and d), suggesting the induction of these compounds may also contribute to plant defense (Fig. 6f).

Additionally, other phytohormones like cytokinin were originally identified as a regulator of plant cell division. Recently, various functions of cytokinin in plant response to abiotic or biotic stresses have been reported [35]. Cytokinin has been demonstrated to play an important role in plant immune response [36]. Increased levels of cytokinin such as trans-zeatin induced resistance against mainly biotrophic or hemi-biotrophic pathogens in different plant species [37]. Here, upon *C. camelliae* Δ*CcCp1* infection, trans-zeatin was increased compared with CCA-infected Longjing 43 and control (Figs 5b and 6b; Fig. S16, see online supplementary material). This indicated *C. camelliae* CCA might suppress trans-zeatin accumulation in tea plant by the cerato-platanin protein CcCp1 (Fig. 6f).

## Discussion

Tea plant contains high levels of anti-fungal compounds which provide plants with basic resistance towards tea pests [1, 5, 15]. However, tea plants are now attacked by some insects and fungi [2]. Thus, tea pests have evolved a strategy to overcome tea plant defense responses and benefit themselves. Recently, the small secreted cysteine-rich proteins, cerato-platanin proteins, are reported to contribute to fungal pathogenicity and have a function in plant–pathogen interaction [24, 44]. In this work, we observed one cerato-platanin protein, CcCp1, involved in tea plant–*C. camelliae* interaction and expression of *CcCp1* was increased during fungal infection. Similarly, expression of *SsCP1* was increased in *S. sclerotiorum* during interaction with *Arabidopsis thaliana* at an early stage [28]. Differently, *B. cinerea BcSpl1* showed variable levels of expression upon interaction with tomato [25]. The amount of *BcSpl1* mRNA increased with a two-phase curve during the interaction: the lower peak presented during conidia germination and the initial black infection spot formation, and the highest peak was observed when the necrosis lesions were expanding at late stages of infection [25].

Several works had proved the members of cerato-platanin protein family were associated with fungal hyphal growth and chlamydospores formation [38–40]. For example, the *C. gloeosporioides* CgCP1 contributed to fungal conidiation and plays a role in rubber tree–pathogen interaction [40]. Conidiation of ∆*CgCP1* significantly decreased when it was compared with the wild-type strain [40]. However, the conidiation was not affected in other works such as *B. cinerea* ∆*BcSpl1* [25]. Our work indicated *C. camelliae* CcCp1 together with *C. gloeosporioides* CgCP1 contributed to the ability to produce conidia, which might play a special role during interaction with plants. In addition, several cerato-platanin proteins also contributed to fungal virulence such as *B. cinerea* BcSpl1, *C. gloeosporioides* CgCP1, *S. sclerotiorum* SsCP1, *Magnaporthe oryzae* MSP1, etc [25, 28, 40, 41]. *C. camelliae* CcCp1 together with these CPs played a similar role in fungal virulence.

Plants attacked by fungal pathogens would trigger transcriptional reprogramming and metabolic changing in both sides. Here, upon *C. camelliae* CCA treatment, tea plant transcripts changed including genes associated with hormones biosynthesis and transduction, secondary metabolism, plant-pathogen interaction, transcription factors, etc. Lost function of gene mutants (∆*CcCp1*) altered above genes’ expression levels indicating *C. camelliae CcCp1* involved in affecting tea plant defense genes’ expression. As a result, tea plant metabolites changed, especially the metabolites in the lipid pathway. In a previous study, Wang *et al.*, tested the transcription differences between the resistant and susceptible tea cultivars towards *C. fructicola* and observed 3007 DEGs enriched in 23 pathways in the resistant cultivar, of which phenylalanine metabolism and flavonoid biosynthesis were markedly enriched [42]. The key genes associated with disease resistance were enriched in transcription factors, secondary metabolites, and cell death [42]. Based on the microarray data of tea plant different cultivars towards *C. camelliae*, it indicated that the DEGs are mapped to phenylpropanoid biosynthesis, phenylalanine metabolism, and flavonoid biosynthesis pathway [6]. Recently, Lu *et al.* performed transcriptomic and metabolomic analyses of the wounded leaves of tea cuttings upon *C. camelliae* LS_19 infection revealing that levels of phytohormones like JA and IAA biosynthesis were increased [43]. However, differentially, the accumulation of polyunsaturated fatty acid and IAA were observed at a late stage (72 hpi) when disease spots appeared [43]. Here, the differentially accumulated metabolisms observed in our work are perhaps due to the different treatments and different isolates used.

Previous works revealed most CPs are secreted, which localized at the fungal cell wall, indicating their active function in host–fungi interaction [44]. CPs were reported to facilitate the infection of virulent fungi either by helping them inside the plant, or by promoting the access of fungal virulence factors into the host cell, or by loosening of plant cell wall materials, or by enabling the acquisition of nutrients after cell death [44]. Furthermore, *B. cinerea* BcSpl1 bound to plant plasma membranes and caused cell shrinkage and chloroplast disorganization, thus resulting in the observed necrosis [27]. We observed that the tea plant lipid pathway (e.g. linolenic acid and linoleic acid metabolism, glycerophospholipid metabolism, etc) was unexpectedly affected by *C. camelliae* CCA compared with ∆*CcCp1*, suggesting *C. camelliae* CcCp1 played a key role in promoting disease by modulating plant lipid metabolism.

Because certain metabolites had antifungal activity, the pathogen might target these compounds [16]. For example, *Ustilago maydis* effector Tin2 was required for both fungal virulence and the biosynthesis of plant anthocyanin [45]. Tin2 involved in the increase of anthocyanin, negatively affected the lignin biosynthetic pathway [45]. This prevented lignification of the plant cell wall, which would enhance a physical barrier to pathogen spread [45]. The fungal *QDO* genes encoding quercetin dioxygenase were reported to degrade flavonoids such as quercetin and kaempferol, while the gene mutants partly lost their function in chemical degradation and showed lower virulence in both *B. cinerea* and *S. sclerotiorum* [16, 46]. More recently, a *CcUOX* gene encoding urate oxidase from *C. camelliae* was indicated to be involved in fungal tolerance towards caffeine and contributed to fungal virulence [47]. Therefore, suppressing of the antifungal metabolism biosynthesis (e.g. EGCG, caffeine) could be one of the strategies for *C. camelliae* to cause disease. Whether CcCp1 protein was involved in manipulating antifungal compound degradation needs further investigation.

Another strategy for fungal virulence was by modulating host hormones crosstalk. Different to the induction of jasmonic acid in Longjing 43 upon *C. camelliae* CCA infection, ∆*CcCp1*-treated tea plant Longjing 43 accumulated cytokinin such as trans-zeatin. It seemed to be part of the pathogen infection strategy because stabilization of cytokinin levels enhanced plant resistance against the fungus. Similarly, after infection by the hemi-biotrophic fungus *Verticillium longisporum*, the trans-zeatin levels in *Arabidopsis* were decreased [48]. The defense responses of cytokinin may be dependent or independent of the SA pathway [35]. In rice–*M. oryzae* interaction, the fungus expressed cytokinin synthesis 1, which contributed to fungal virulence [49]. After *M. oryzae* infection, the cytokinin and SA worked together to induce defense gene expression depending on the SA receptor OsNPR1 and the TF WRKY45 [50]. Other studies of cytokinin in plant defense responses were those against *Pseudomonas syringae* pv. tomato DC3000 (Pst) or *Hyaloperonospora arabidopsidis* (Hpa) Noco2 [36]. Cytokinin was also involved in plant–herbivore interactions. It indicated JA pathway was not required to increase the cytokinin levels but even suppressed the cytokinin responses [36]. Here, *C. camelliae* CcCp1 was very likely involved in tea plant jasmonic acid–cytokinin crosstalk during compatible interaction and needs further elucidation.

## Materials and methods

### Tea plant, fungi material, and treatments

Tea plant cultivars Longjing 43 (LJ), Zhongcha 108 (ZC), Jinxuan, Guiding, Beishan, and Hongyan 12 were used and two-year-old plants were kept in a microbe-free climate chamber (PGX-600C, Saifu, China). *C. camelliae* indicated strains were grown on PDA plate and the infection was performed as previously [3, 4]. For tea plant mRNA sequencing, the spores were spray incubated on healthy, undetached Longjing 43 and Zhongcha 108 for 24 hours and then tea leaves harvested. For control, tea leaves were only incubated with ddH_2_O. 3-independent biological replicates were performed. For tea plant metabolite analysis, the same treatments were performed and tea leaves were collected with 6-biological replicates. All samples were frozen at −80°C until used.

### Constructing the *C. camelliae* CcCp1 deletion and complementation strains

Flanking DNA sequences of *CcCp1* were cloned into the PXEH replacement vector as indicated previously [16]. The replacement vector was transformed into *C. camelliae* CCA by *Agrobacterium tumefaciens* AGL1 [16]. After screening on selective media, the transformants were confirmed by PCR and qPCR methods (Table S6, see online supplementary material). To get the complementation strains, a PCR fragment containing CcCp1 gene was cloned and transformed into △*CcCp1* mutant spores as previously [16].

### RNA sequencing, fragments mapping, and quantification of gene levels

RNA isolation, purification, and monitoring; cDNA library construction and sequencing; raw data cleaning and analysing were done as indicated previously (LC-Bio, Technology Co., Ltd, China) [16].

Reference genome and gene model annotation files were downloaded from the website [51] (https://ngdc.cncb.ac.cn/search/?dbId=gwh&q=GWHACFB00000000). All sequence data was submitted to the NCBI with accession numbers GSE205689/208559. An index of the reference genome was built, paired-end clean reads were then aligned to the reference, and the FPKM of each gene was finally calculated as performed previously [16].

### Analysis of differentially expressed genes

DEGs in all samples (CK, CCA, △*CcCp1*) were performed as previously [16]. DEGs were identified by the R package.

### Gene ontology and Kyoto Encyclopedia of Genes and Genomes enrichment analysis of DEGs

Gene Ontology (GO) enrichment and Kyoto Encyclopedia of Genes and Genomes (KEGG) analysis of DEGs were performed as previously [16].

### Metabolites extraction and parameters setting

For extraction of plant metabolites, 20 μl of leaf sample was extracted with 120 μl of precooled 50% methanol buffer as indicated previously [16]. All leaf samples were analysed by the Liquid Chromatograph Mass Spectrometer (LC–MS) system according to machine orders (LC-Bio, Technology Co., Ltd, China) [16].

### Identification and quantification of tea plant metabolites

LC–MS raw data was fist transformed into the mzXML format by using MSConvert, and then processed by the XCMS, CAMERA, and metaX toolbox, implemented in the R software [16]. The combined retention time (RT) and m/z data were used to identify each ion.

## Acknowledgements

This work was partly supported by the National Natural Science Foundation of China (NSFC grant No. 32171801 to S.L.) and the Cross-Disciplinary Innovation Founding of Jilin University No. JLUXKJC2020313 (S.L.).

## Author contributions

S.L. designed the research plan and wrote the article. S.Z., S.H., X.Q., R.A., and S.L. performed the research.

## Data availability

All data needed to evaluate the conclusions in the paper are present in the paper and/or the Supplementary Materials.

## Conflict of interest statement

The authors declare that they have no conflict of interest.

## Supplementary data

Supplementary data is available at *Horticulture Research* online.

## Supplementary Material

Web_Material_uhad028
