# Supplementary material for: Tea plant (Camellia sinensis) lipid metabolism pathway modulated by tea field microbe (Colletotrichum camelliae) to promote disease
Source: Hortic Res. 2023 Feb 21;10(4):uhad028. doi: 10.1093/hr/uhad028 (PMC10117433; doi:10.1093/hr/uhad028)
Supplement: Web_Material_uhad028 [file web_material_uhad028.zip › Figure S 20230123.pdf]

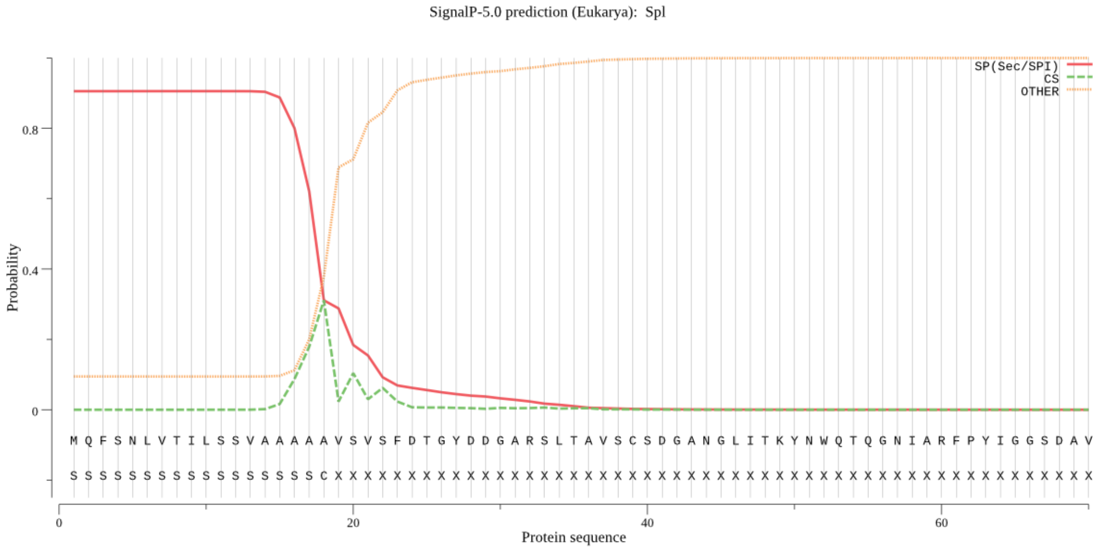

Fig.S1. SignalP prediction of cleavage site of CcCp1.

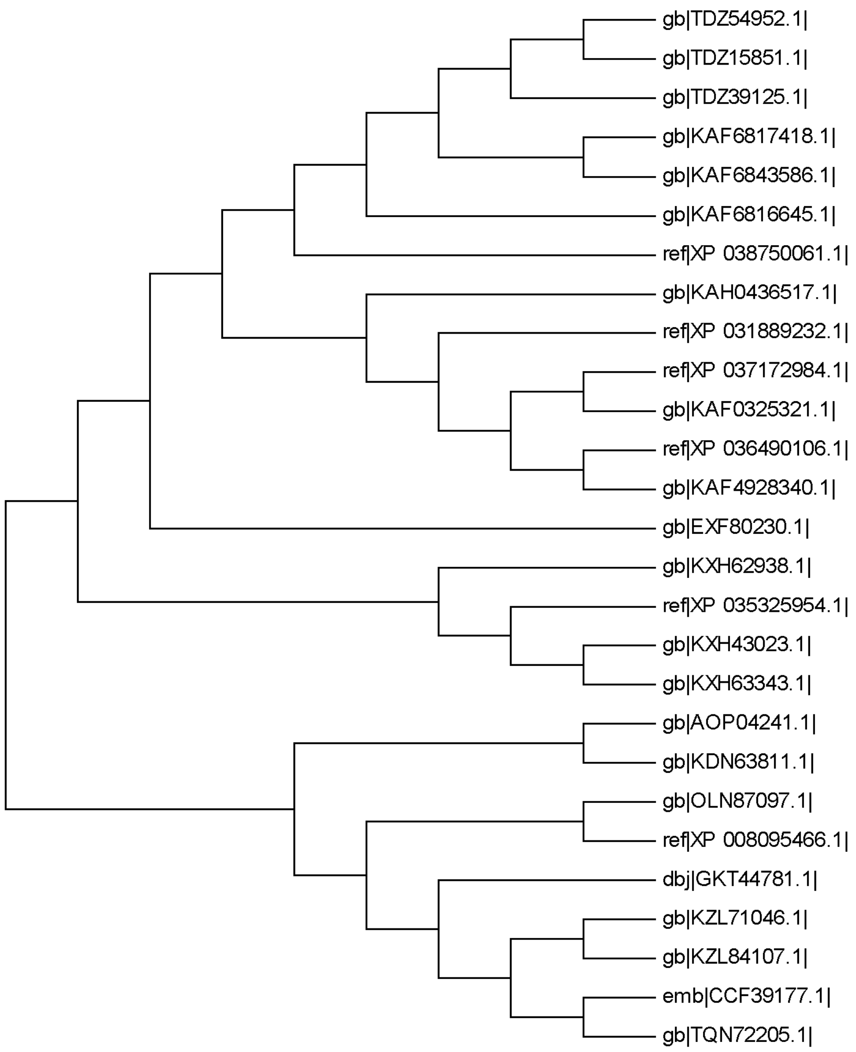

Fig.S2. Phylogenetic tree of CcCp1 protein and the indicated proteins. These proteins including *Colletotrichum trifolii* TDZ54952.1, *C. orbiculare* MAFF 240422 TDZ15851.1, *C. spinosum* TDZ39125.1, *C. plurivorum* KAF6817418.1, *C. musicola* KAF6843586.1, *C. sojae* KAF6816645.1, *C. karsti* XP_038750061.1, *C. camelliae* KAH0436517.1, *C. fructicola* XP_031889232.1, *C. aenigma* XP_037172984.1, *C. asianum* KAF0325321.1, *C. siamense* XP_036490106.1, *C. viniferum* KAF4928340.1, *C. fioriniae* PJ7 EXF80230.1, *C. nymphaeae* SA-01 KXH62938.1, *C. scovillei* XP_035325954.1, *C. simmondsii* KXH43023.1, *C. salicis* KXH63343.1, *C. falcatum* AOP04241.1, *C. sublineola* KDN63811.1, *C. chlorophyti* OLN87097.1, *C. graminicola* M1.001XP_008095466.1, *C. liriopes* GKT44781.1, *C. tofieldiae* KZL71046.1, *C. incanum* KZL84107.1, *C. higginsianum* CCF39177.1, *C. shisoi* TQN72205.1.

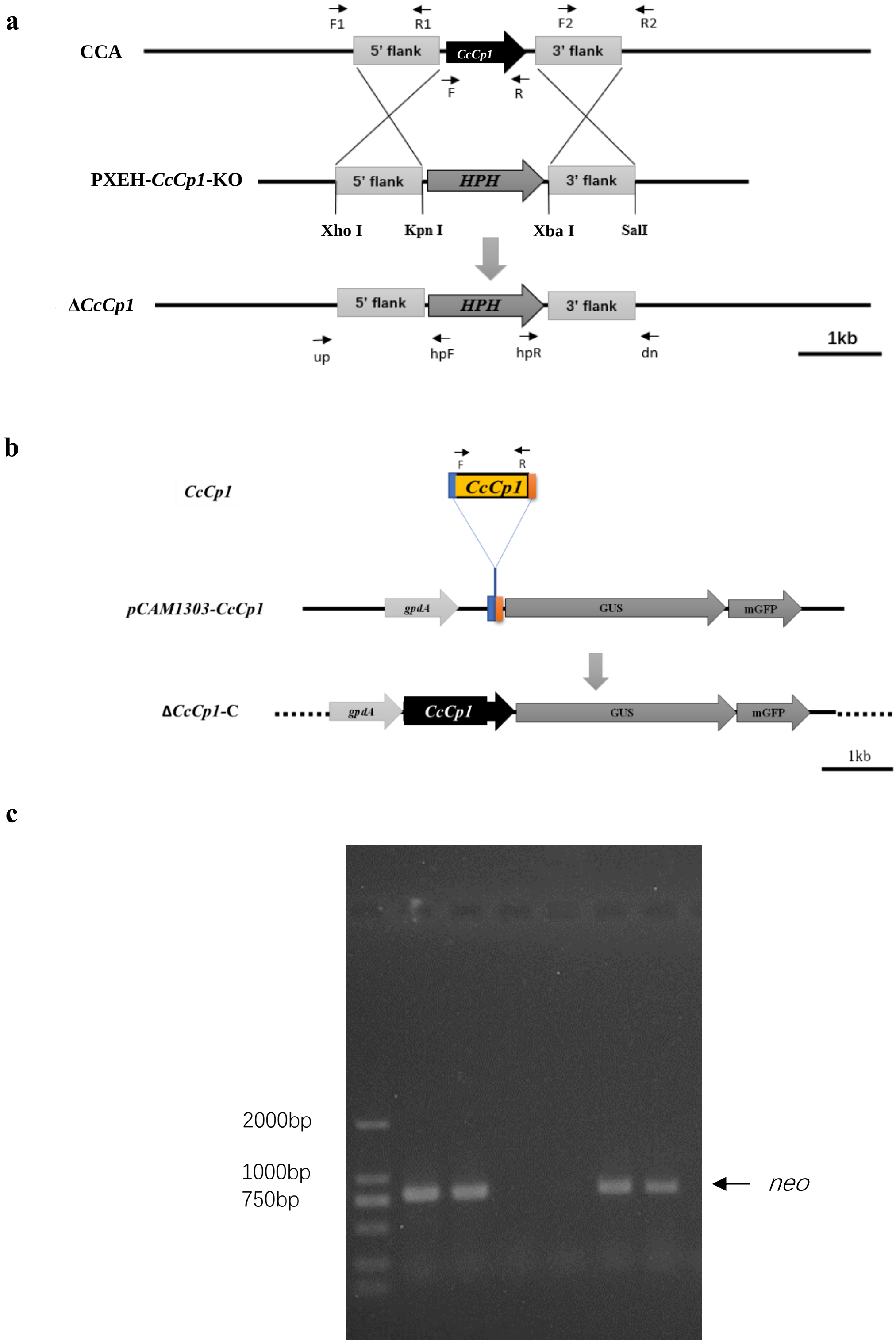

Fig. S3. Construction, confirmation and pathogenic analysis of Δ*CcCp1*-C complement line. **a** Strategy for construction of *CcCp1* gene deletion mutants. **b** Strategy for construction of Δ*CcCp1*-C complement strain. **c** PCR confirmation of Δ*CcCp1*-C complement strains. The neo gene was amplified by PCR method.

a

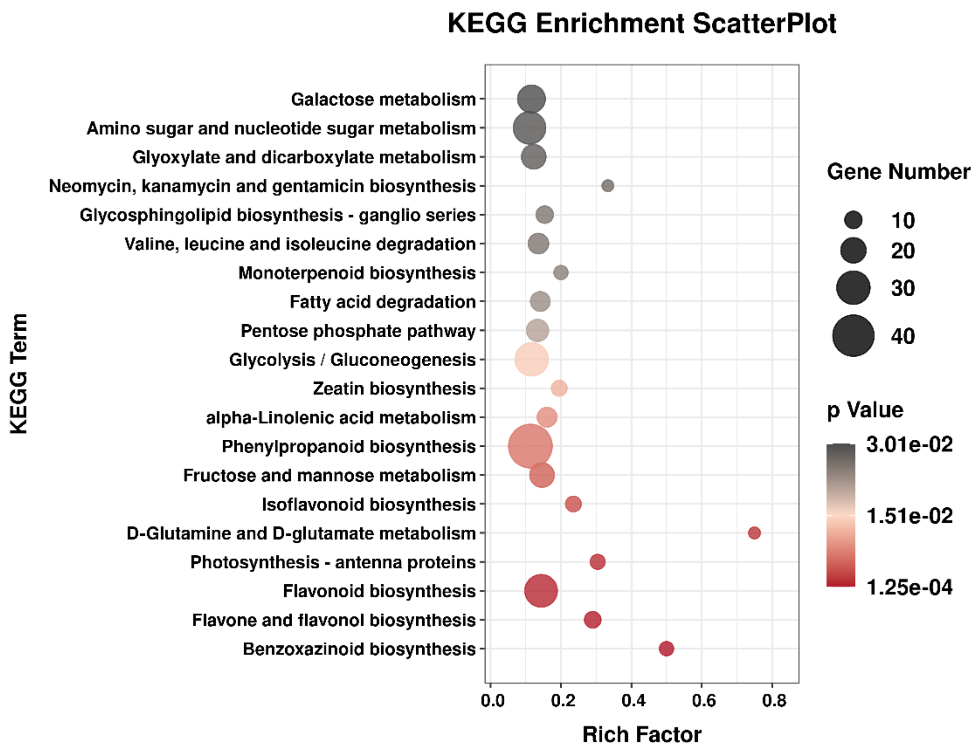

b

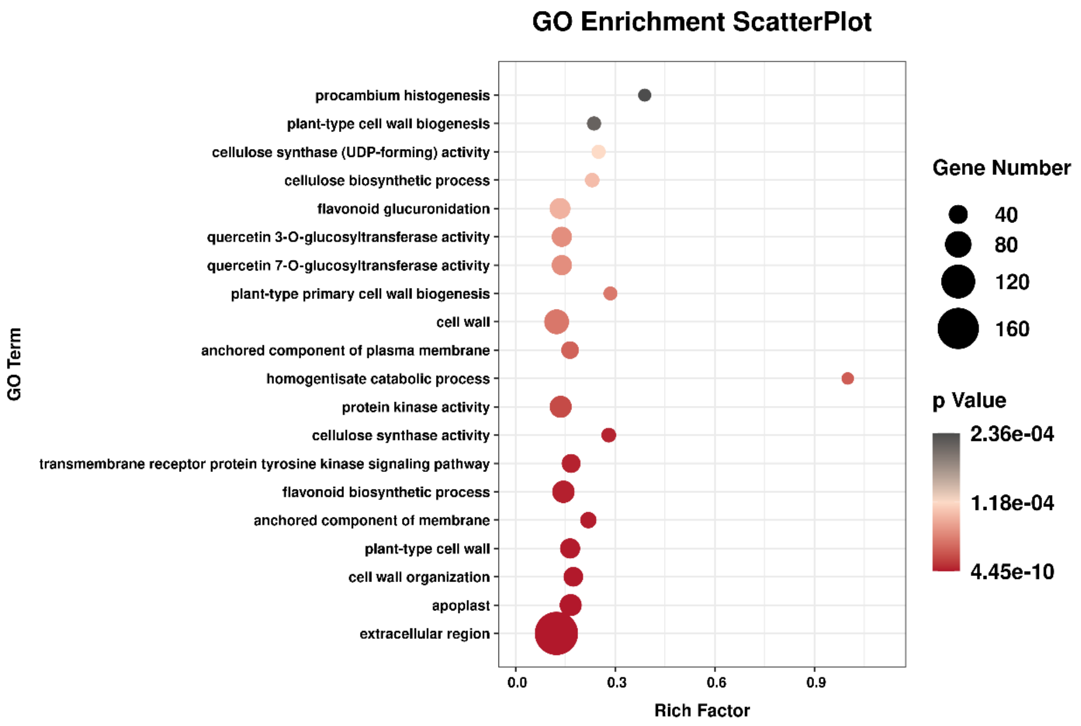

Fig. S4. KEGG and GO analysis of differentially expressed genes in tea plant Longjing 43. a KEGG analysis of differentially expressed genes in tea plant Longjing43 upon *C. camellia* CCA and Δ*CcCp1* infection. **b** GO analysis of differentially expressed genes in tea plant Longjing43 upon *C. camellia* CCA and Δ*CcCp1* infection.

a

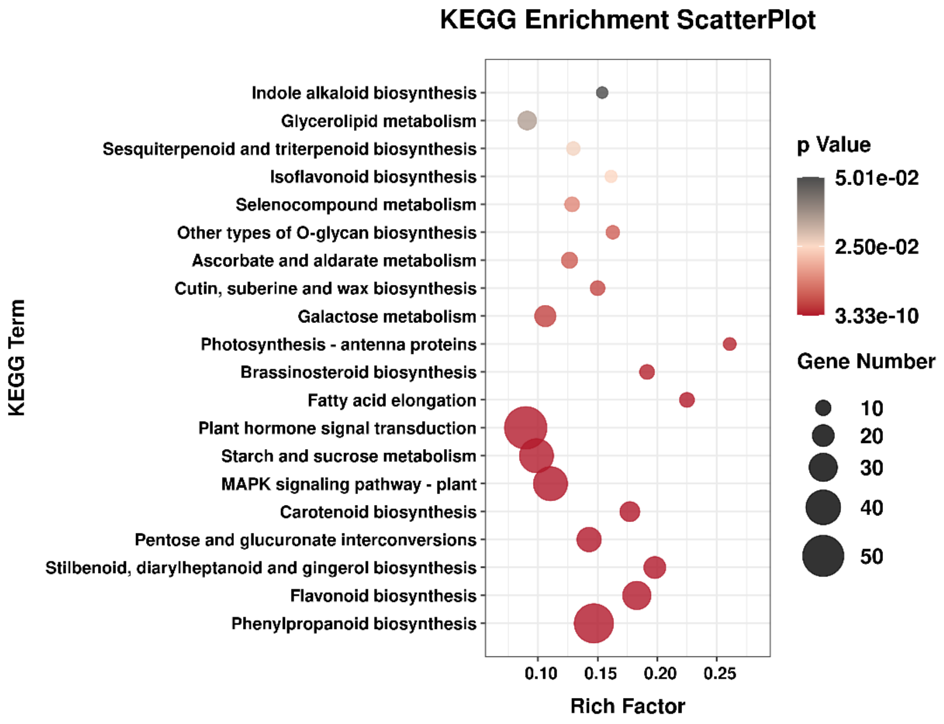

b

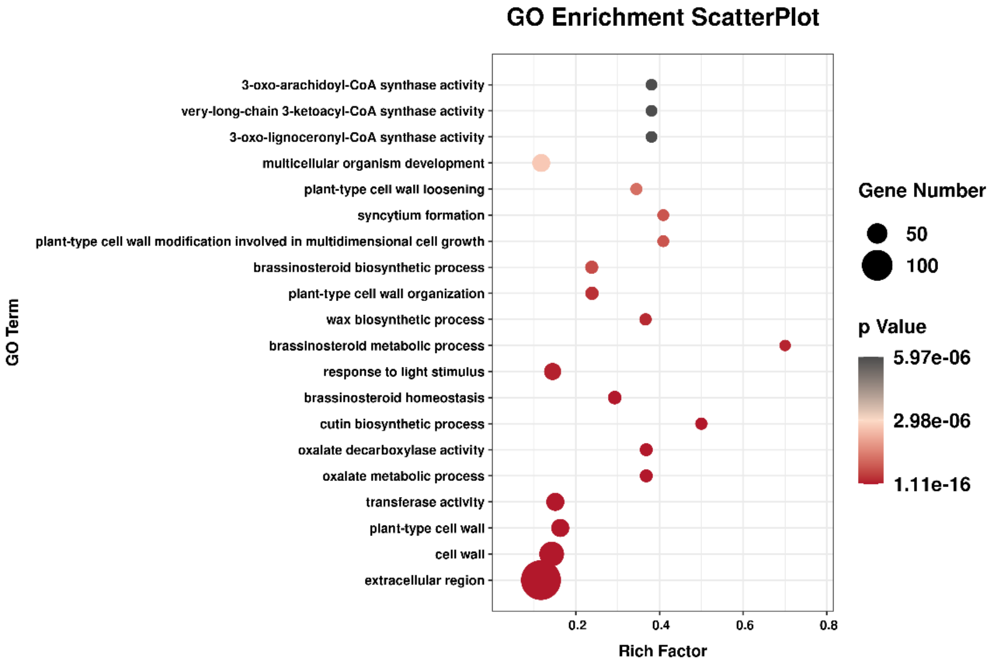

Fig. S5. KEGG and GO analysis of differentially expressed genes in tea plant Zhongcha 108. a KEGG analysis of differentially expressed genes in tea plant Zhongcha108 upon *C. camellia* CCA and Δ*CcCp1* infection. **b** GO analysis of differentially expressed genes in tea plant Zhongcha108 upon *C. camellia* CCA and Δ*CcCp1* infection.

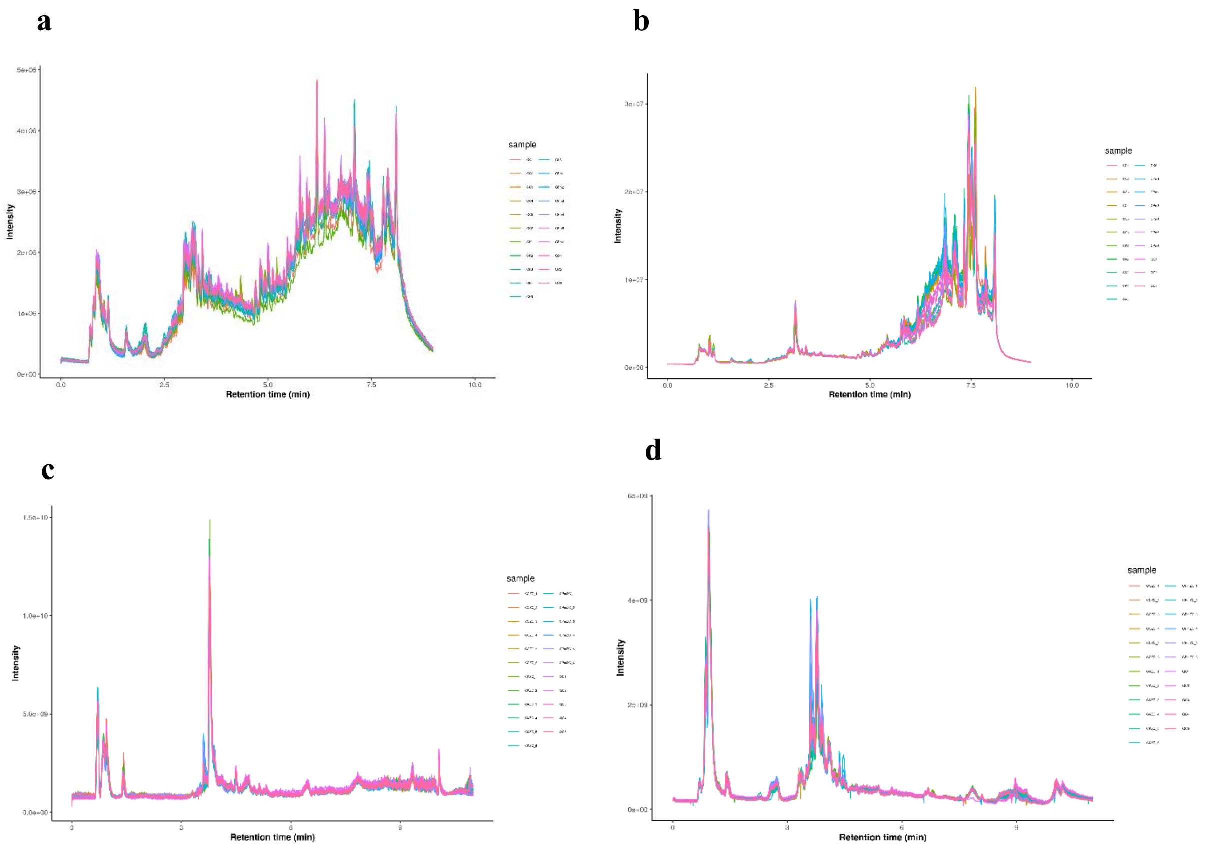

Fig. S6. The total ion chromatograms of all the samples. Total ion chromatogram in POS model and NEG model during tea plant Longjing 43 (**a, b**) or Zhongcha 108 (**c, d**) interaction with *C. camellia* CCA and Δ*CcCp1*, respectively. The x-axis represents retention time and the y-axis represents the sum of intensity for all the ions in MS. The overlaps of QC samples in TIC can be used to preliminarily judge the state of the instrument. The higher intensity overlaps present, the more stable the instrument is.

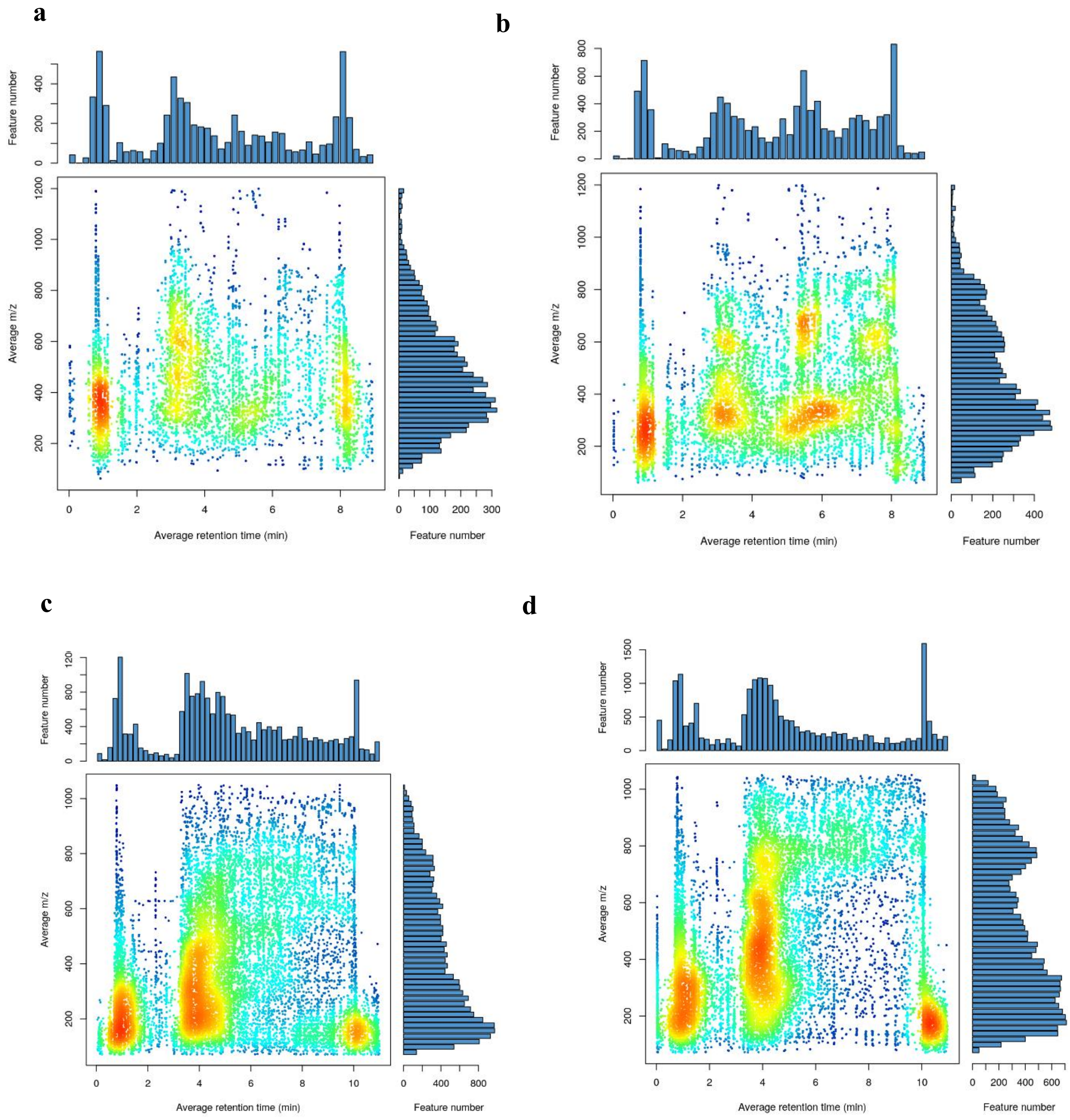

Fig. S7. Evaluation of average retention time and average m/z for identified features in Longjing 43 and Zhongcha 108. a POS model, Longjing 43; **b** NEG model Longjing 43; **c** POS model, Zhongcha 108; **d** NEG model Zhongcha 108. The x-axis represents the average retention time and the y-axis represents the average m/z. The color depth indicates feature intensity.

a

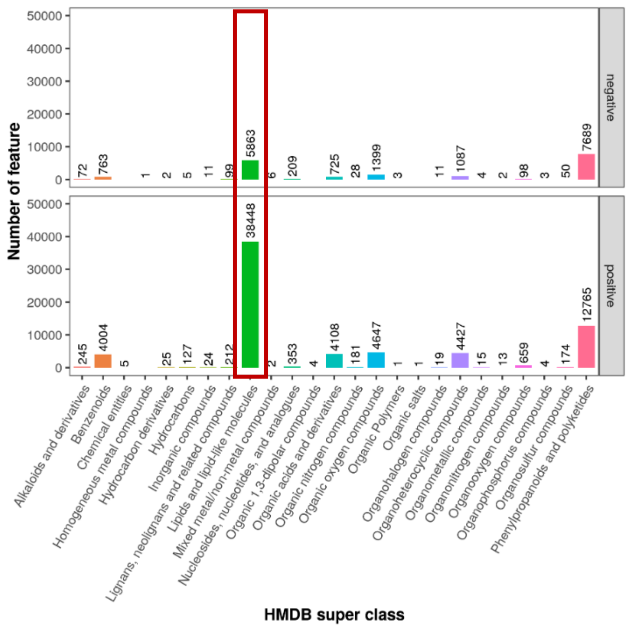

b

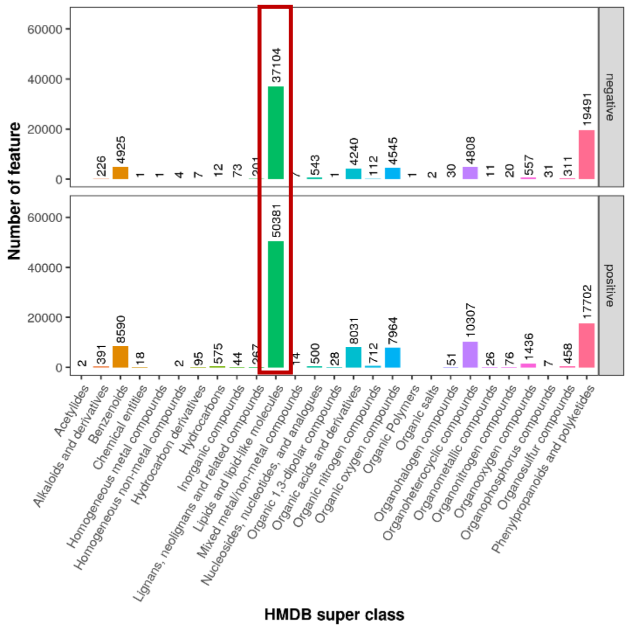

Fig. S8. The metabolites observed in tea cultivars assigned to the HMDB super class. **a** The metabolites observed in Longjing 43 assigned to the HMDB databases and the framed indicates the features enriched in lipids and lipid-like molecules. **b** The metabolites observed in Zhongcha 108 assigned to the HMDB databases and the framed indicates the features enriched in lipids and lipid-like molecules.

a

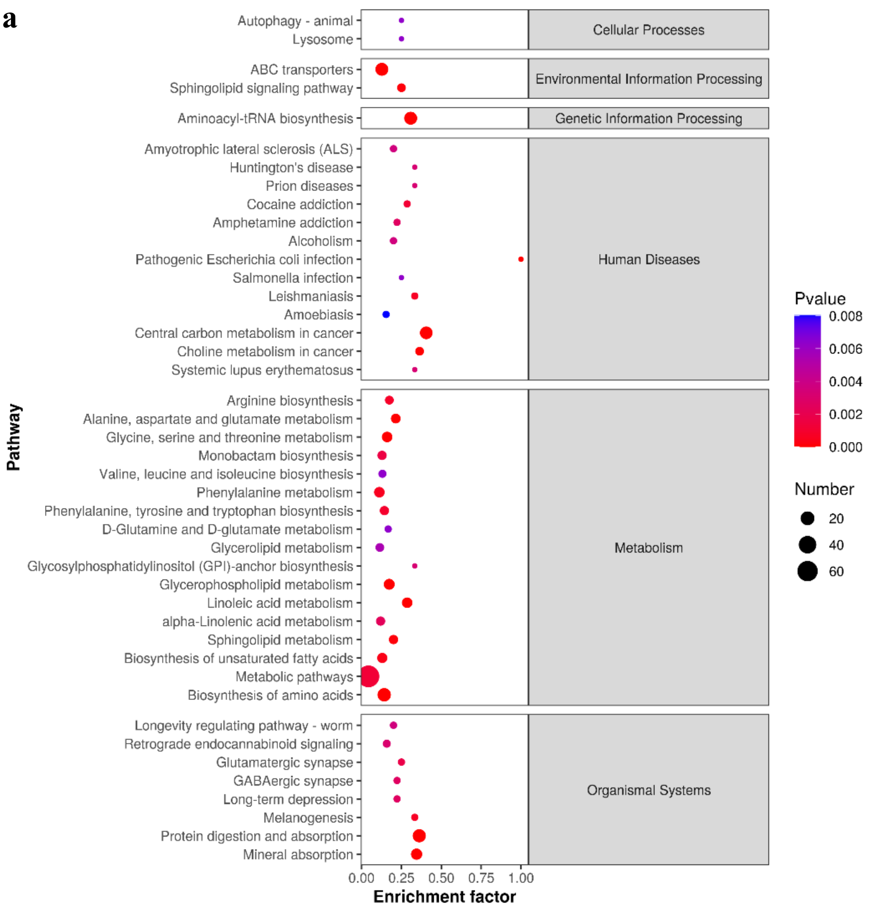

b

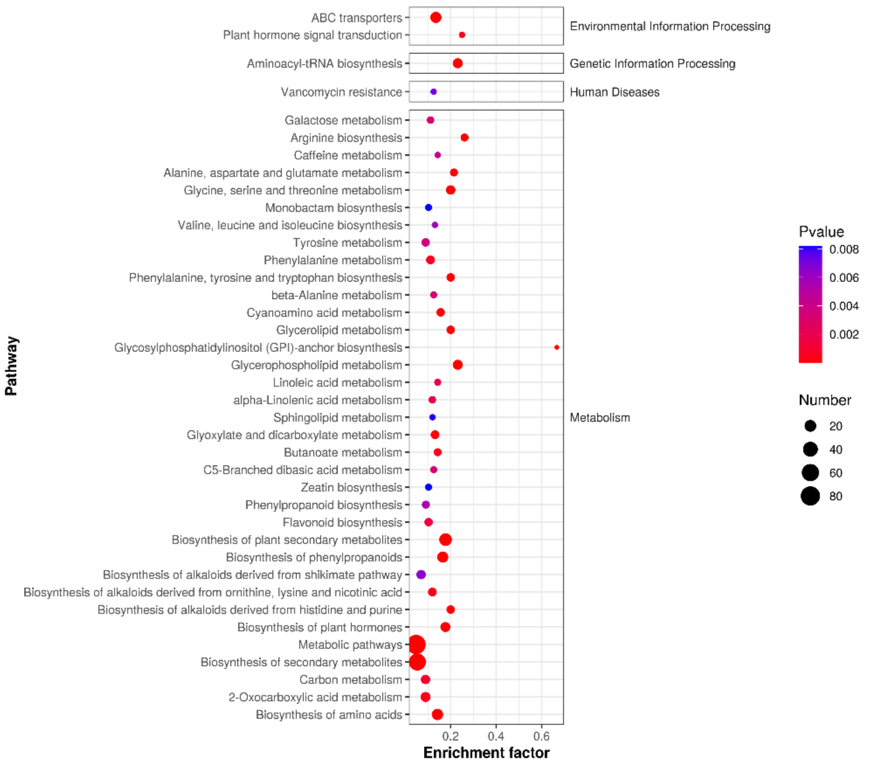

Fig. S9. Level-3 terms of the KEGG pathway classification, metabolites detected

and annotated. a Longjing 43; **b** Zhongcha 108.

a

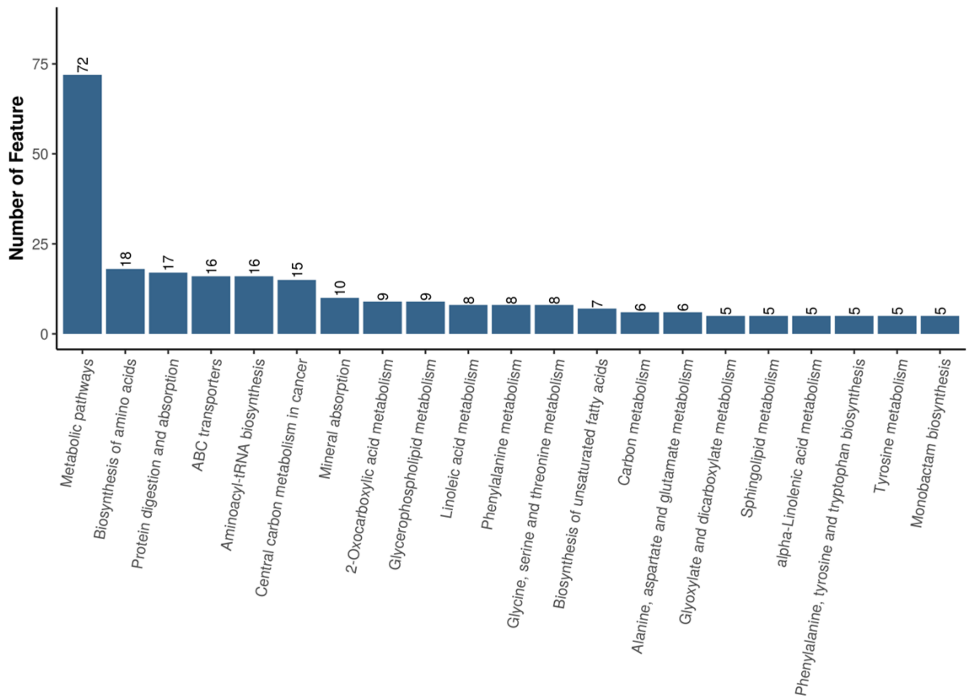

b

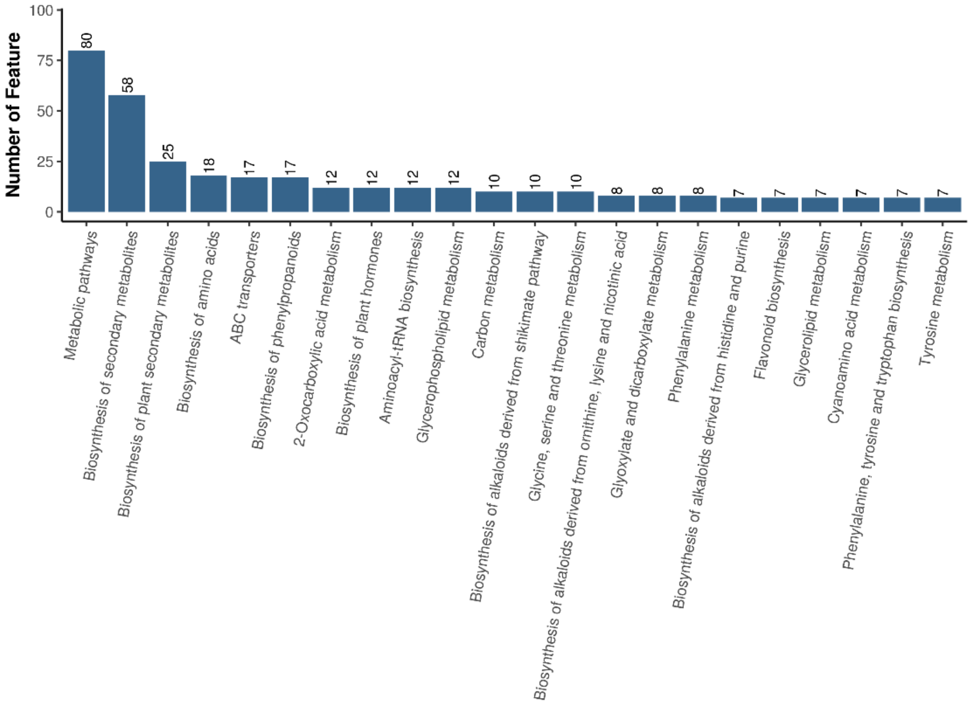

Fig. S10. All identified metabolites classified into the top KEGG pathways. The x-axis represents the top KEGG pathways and the y-axis represents number of identified metabolites involved in this pathway. **a** Longjing 43; **b** Zhongcha 108.

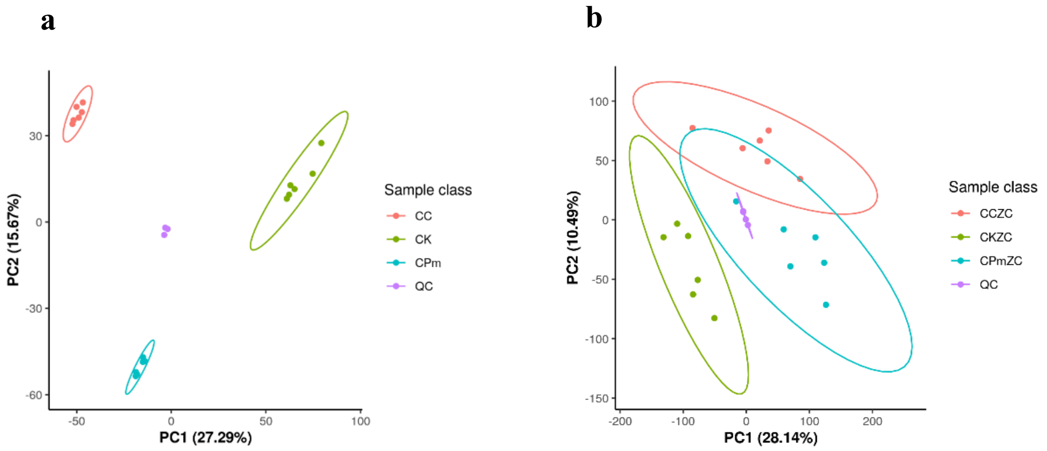

Fig. S11. The principal component analysis of the data from CK and *C. camelliae* CCA and Δ*CcCp1* infected tea plants. PC1 represents the first principal component; PC2 represents the second principal component. CK, control; CC, *C. camelliae* CCA treated plants; CPm, *C. camelliae*Δ*CcCp1* treated plants; QC, quality control (a mixture of experimental samples prepared in equal amounts). The data shown are the means of six biological replicates. **a** Longjing 43; **b** Zhongcha 108.

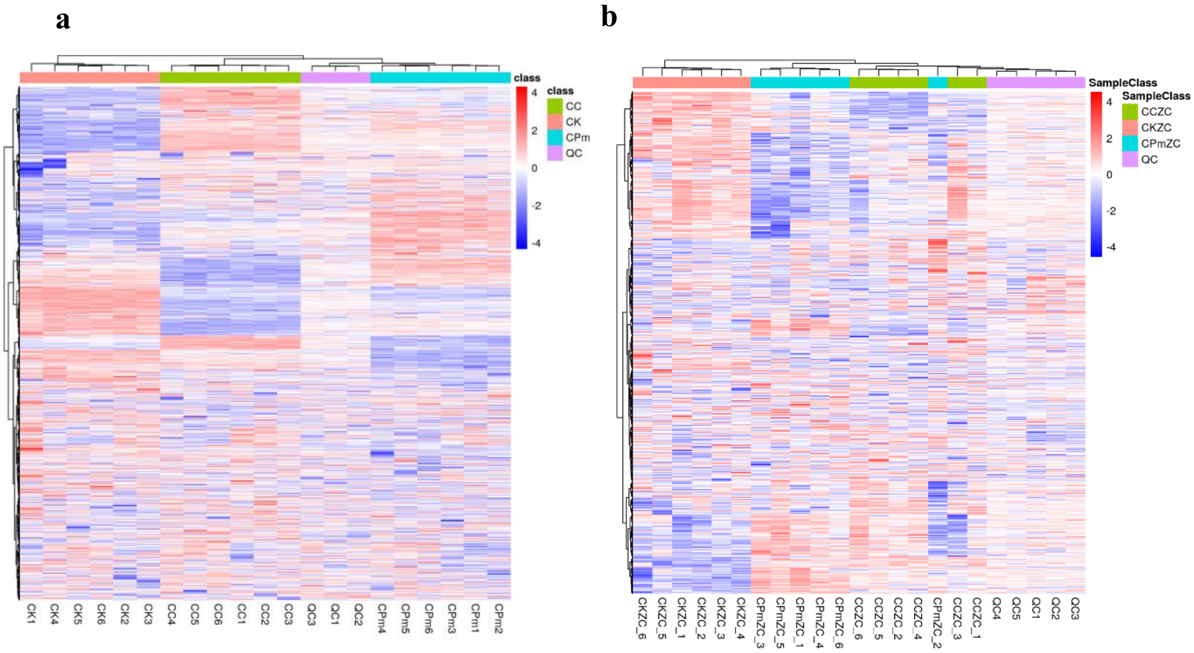

Fig. S12. Heatmap of the metabolites identified in the metabolome of mock treatment (CK) or *C. camelliae* CCA treated tea plants (CC) or Δ*CcCp1* treated tea plants (n=18). The heatmap scale ranges from −4 to +4 on a $\log_2$ scale. **a** Longjing 43; **b** Zhongcha 108.

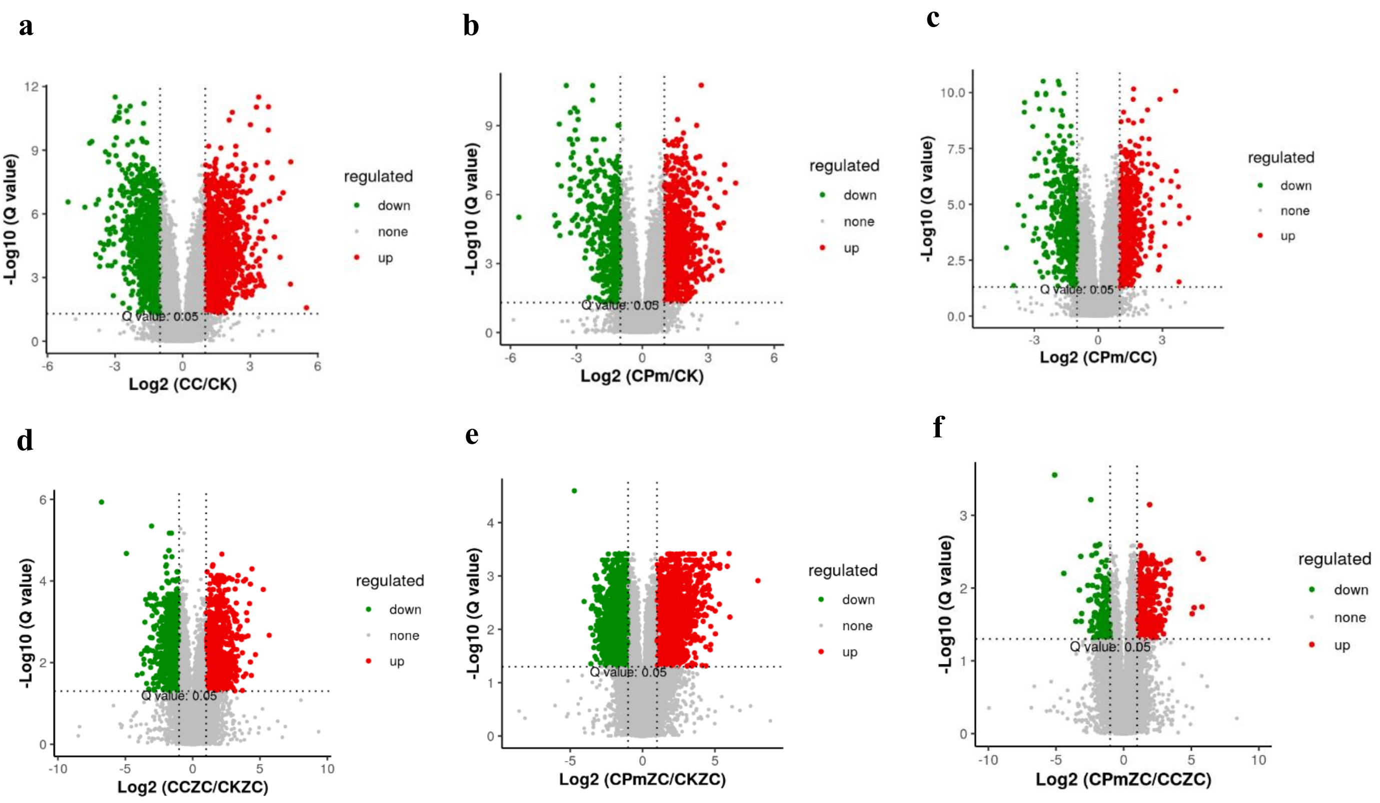

Fig. S13. Identification of the differentially accumulated metabolites (DAMs) between CK, *C. camelliae* CCA and Δ*CcCp1* infected tea plants Longjing43 (a-c) and Zhongcha 108 (d-f). Significance analysis of the DAMs between the treatments by Volcanoplot. **a, d** *C. camelliae* CCA vs CK; **b, e** *C. camelliae*Δ*CcCp1* vs CK; **c, f** *C. camelliae* Δ*CcCp1* vs CCA.

a

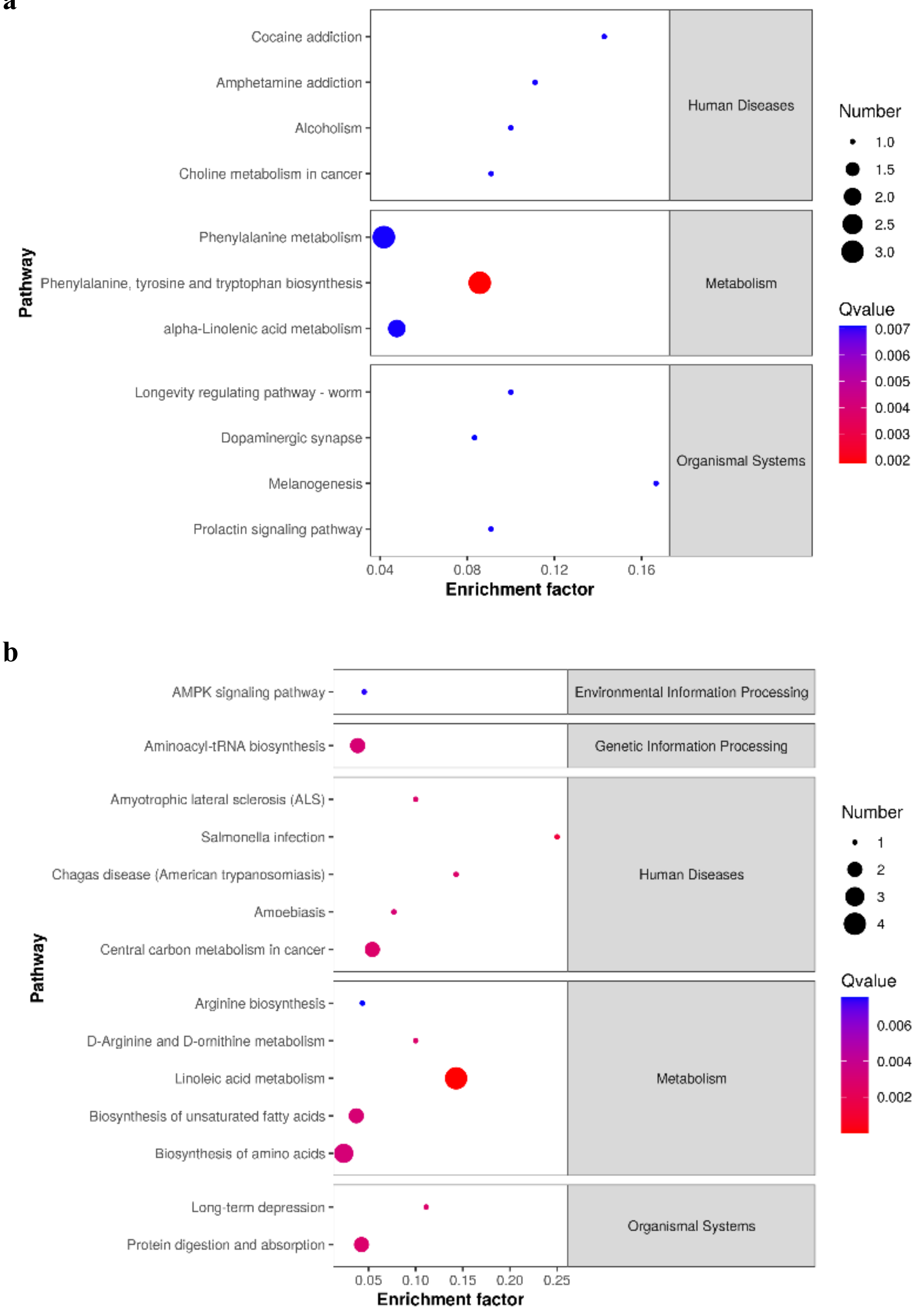

Fig. S14. KEGG analysis of differentially accumulated metabolites (DAMs) between CK, *C. camelliae* CCA and Δ*CcCp1* infected tea plants Longjing 43. a *C. camelliae* CCA vs CK; **b** *C. camelliae* Δ*CcCp1* vs CK.

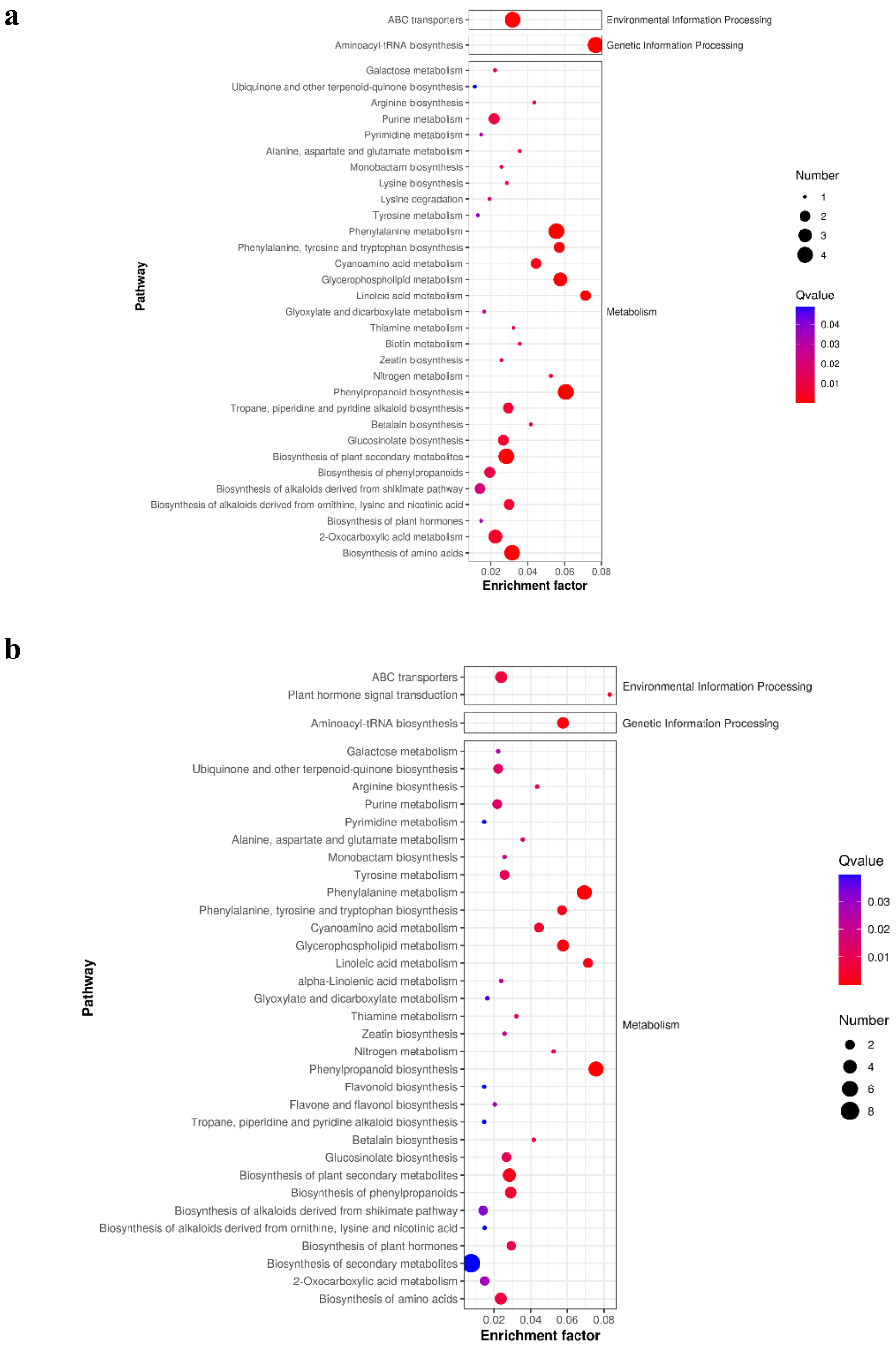

Fig. S15. KEGG analysis of differentially accumulated metabolites (DAMs) between CK, *C. camelliae* CCA and Δ*CcCp1* infected tea plants Zhongcha108. a *C. camelliae* CCA vs CK; **b** *C. camelliae* Δ*CcCp1* vs CK.

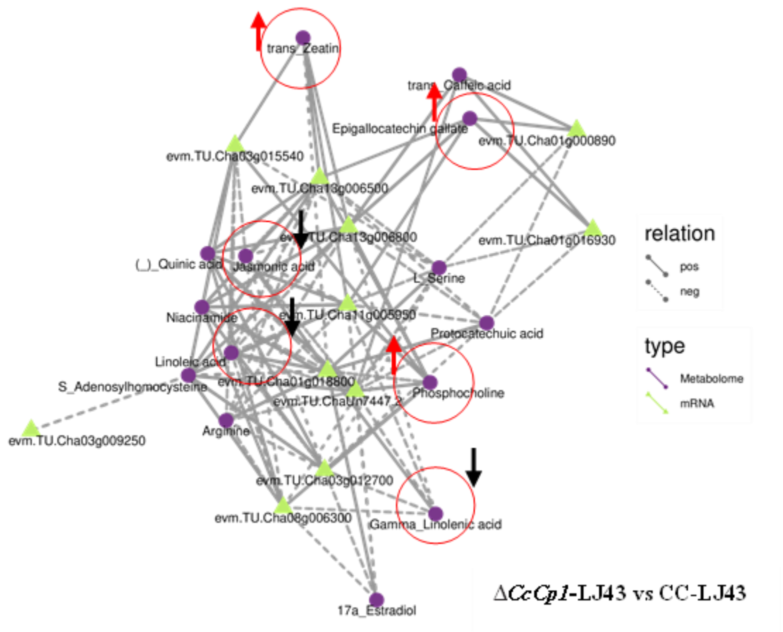

Fig. S16. The putative genetic and metabolic regulatory networks between *C. camelliae* Δ*CcCp1* and CCA treated tea plant Longjing 43. The arrows indicated the comparison of metabolite changes in tea plats upon Δ*CcCp1* and CCA infection (red arrow: increased; black arrow: decreased).
