# Supplementary material for: Tea plant (Camellia sinensis) lipid metabolism pathway modulated by tea field microbe (Colletotrichum camelliae) to promote disease
Source: Hortic Res. 2023 Feb 21;10(4):uhad028. doi: 10.1093/hr/uhad028 (PMC10117433; doi:10.1093/hr/uhad028)
Supplement: Web_Material_uhad028 [file web_material_uhad028.zip › Table S20221220.pdf]

Table S1 Total validated high-quality reads were obtained from all libraries.

Sample	Raw Data		Valid Data		Valid Ratio(reads)	Q20%	Q30%	GC content%
	Read	Base	Read	Base				
CCLJ1	40301970	6.05G	39374592	5.91G	97.70	99.98	97.74	46
CCLJ2	44165174	6.62G	43311340	6.50G	98.07	99.98	97.55	47
CCLJ3	43888232	6.58G	42990102	6.45G	97.95	99.98	97.68	46
CKLJ1	39240834	5.89G	38337096	5.75G	97.70	99.97	97.74	46.50
CKLJ2	49208936	7.38G	47907670	7.19G	97.36	99.97	97.82	47
CKLJ3	51400032	7.71G	50314074	7.55G	97.89	99.98	97.99	45
CPmLJ1	54048524	8.11G	52272312	7.84G	96.71	99.98	97.60	46.50
CPmLJ2	48083526	7.21G	46720470	7.01G	97.17	99.97	97.47	46
CPmLJ3	53691078	8.05G	52186152	7.83G	97.20	99.97	97.53	46
CCZC1	39842138	5.98G	38858730	5.83G	97.53	99.97	95.63	44
CCZC2	42810000	6.42G	41756826	6.26G	97.54	99.97	95.57	43.50
CCZC3	39132178	5.87G	38187522	5.73G	97.59	99.98	95.91	44
CKZC1	38044738	5.71G	37188678	5.58G	97.75	99.97	95.97	44
CKZC2	41946602	6.29G	40922294	6.14G	97.56	99.97	95.86	43.50
CKZC3	37973358	5.70G	37009050	5.55G	97.46	99.97	95.60	43.50
CPmZC1	47913502	7.19G	46736374	7.01G	97.54	99.97	95.63	44
CPmZC2	40542484	6.08G	39662864	5.95G	97.83	99.97	95.77	43.50
CPmZC3	38980528	5.85G	38061786	5.71G	97.64	99.97	95.67	44

Table S2 Statistics of identified metabolites.

Tea plant	mode	All	Combine intensity	MS2	HMDB	KEGG	Annotated
LJ43	negative	6796	12690	260	2834	2332	3549
	positive	10655		655	6326	5057	7048
ZC108	negative	20643	33729	322	8601	7123	10360
	positive	20345		378	11973	9986	13577

Table S3 Statistics for differential metabolites between different group comparisons.

sample	neg-all	neg-down	neg-up	pos-all	pos-down	pos-up	all regulated
CCLJ/CKLJ	901	492	409	1389	561	828	2290
CPmLJ/CCLJ	544	263	281	678	304	374	1222
CPmLJ/CKLJ	643	310	333	962	350	612	1605
CCZC/CKZC	1287	650	637	918	403	515	2205
CPmZC/CCZC	226	137	89	361	250	111	587
CPmZC/CKZC	2636	1003	1633	1927	674	1253	4563

Table S4 The comprehensive analysis revealed the metabolites associated with lipid pathway are enriched in tea plant Longjing 43 - *C. camelliae* interaction.

Pathway	diff_gene	number of diff_gene	diff_metabolite	number of diff_metabolite
**CCLJ vs CKLJ**				
00592(alpha-Linolenic acid metabolism)	evm.TU.Cha09g007020;evm.TU.ChaUn15007.1;evm.TU.Cha09g007060;evm.TU.Cha11g002480;evm.TU.Cha01g013650;evm.TU.Cha11g002840;evm.TU.Cha09g007070;evm.TU.Cha05g004980;evm.TU.ChaUn5723.1;evm.TU.Cha09g012920;evm.TU.Cha14g012310;evm.TU.Cha09g007080;evm.TU.Cha01g021530;evm.TU.Cha11g002550;evm.TU.Cha06g019480	15	**Jasmonic acid**; **Traumatin**	2
00591(Linoleic acid metabolism)	evm.TU.Cha15g004830;evm.TU.ChaUn15007.1;evm.TU.Cha11g002480;evm.TU.Cha11g002840;evm.TU.Cha14g012310;evm.TU.Cha03g004920	6	**9,12,13-TriHOME**	1
00564(Glycerophospholipid metabolism)	evm.TU.Cha08g013310;evm.TU.ChaUn9054.2;evm.TU.ChaUn7495.1;evm.TU.Cha08g017300;evm.TU.Cha15g006250;evm.TU.Cha13g010510;evm.TU.Cha11g000470;evm.TU.Cha01g020840;evm.TU.Cha11g000480;evm.TU.Cha04g004340;evm.TU.Cha09g000940;evm.TU.Cha08g015690;evm.TU.Cha04g004360;evm.TU.Cha14g012310;evm.TU.ChaU	23	**Phosphocholine;**	1

	n23324.1;evm.TU.Cha15g000640;evm.TU.Cha11g002550;evm.TU.Cha04g006800;evm.TU.Cha10g011350;evm.TU.Cha14g000130;evm.TU.Cha06g007490;evm.TU.Cha09g003730;evm.TU.Cha08g008560;			
**CpmLJ vs CKLJ**				
01040(Biosynthesis of unsaturated fatty acids)	evm.TU.Cha04g021730;evm.TU.ChaUn27389.1;evm.TU.Cha01g013650;evm.TU.Cha04g007900;evm.TU.Cha08g009930;evm.TU.Cha05g004980;evm.TU.Cha13g008220;evm.TU.Cha11g009610	8	**Gamma-Linolenic acid; Linoleic acid**	2
00591(Linoleic acid metabolism)	evm.TU.Cha15g004830;evm.TU.Cha11g002840;evm.TU.ChaUn15007.1;evm.TU.Cha11g002480;evm.TU.ChaUn15301.1;evm.TU.Cha10g001770;evm.TU.Cha03g004920;evm.TU.ChaUn11389.4	8	**13-OxoODE; Gamma-Linolenic acid; Linoleic acid; 9,12,13-TriHOME**	4
**CpmLJ vs CCLJ**				
00592(alpha-Linolenic acid metabolism)	evm.TU.Cha04g021730;evm.TU.Cha14g012310;evm.TU.Cha04g001830;	3	**Jasmonic acid**	1
00591(Linoleic acid metabolism)	evm.TU.Cha03g009250;evm.TU.Cha14g012310;	2	**Gamma-Linolenic acid; Linoleic acid**	2

01040(Biosynthesis of unsaturated fatty acids)	evm.TU.Cha04g021730;evm.TU.ChaUn27389.1;evm.TU.Cha12g012900;evm.TU.ChaUn12706.1;	4	**Gamma-Linolenic acid; Linoleic acid**	2
00564(Glycerophospholipid metabolism)	evm.TU.Cha11g000480;evm.TU.Cha11g000470;evm.TU.ChaUn9054.2;evm.TU.Cha15g000640;evm.TU.Cha08g005170;evm.TU.Cha14g012310;evm.TU.Cha06g007460;evm.TU.Cha06g011130;evm.TU.ChaUn21104.1;	9	**Phosphocholine;**	1

Table S5 The comprehensive analysis revealed the metabolites associated with lipid pathway are enriched in tea plant Zhongcha 108 - *C. camelliae* interaction.

.

Pathway	diff_gene	number of diff_gene	diff_metabolite	number of diff_metabolite
**CCZC vs CKZC**				
00564(Glycerophospholipid metabolism)	evm.TU.Cha15g008840;evm.TU.Cha06g008170;evm.TU.ChaUn6334.6;evm.TU.Cha10g001480;evm.TU.Cha13g001490;evm.TU.Cha15g008470;evm.TU.Cha04g002490;evm.TU.Cha06g007490;evm.TU.Cha12g007270;evm.TU.ChaUn12967.1;evm.TU.Cha02g002080;evm.TU.Cha06g004860;evm.TU.Cha15g000640;evm.TU.Cha04g004360;evm.TU.Cha03g021920;evm.TU.Cha09g006220;evm.TU.Cha01g024670;evm.TU.Cha06g004830;evm.TU.Cha08g008710;evm.TU.Cha11g000470;evm.TU.Cha06g004780;	21	**LysoPE 18:3; LysoPE 18:2; LysoPE 18:1; LysoPC 18:3**	4
**CpmZC vs CKZC**				
00591(Linoleic acid metabolism)	evm.TU.Cha14g012310;evm.TU.ChaUn15007.1;evm.TU.Cha09g011610;	3	**9,10-Dihydroxy-12Z-octadecenoic acid; Alpha-dimorphecolic acid;**	2

00592(alpha-Linolenic acid metabolism)	evm.TU.Cha01g013650;evm.TU.Cha14g012310;evm.TU.ChaUn4917.1;evm.TU.ChaUn15007.1;evm.TU.Cha05g000350;evm.TU.ChaUn28887.1;evm.TU.ChaUn11007.1;evm.TU.ChaUn16077.2;evm.TU.Cha09g011610;	9	**Jasmonic acid;**	1
00564(Glycerophospholipid metabolism)	evm.TU.ChaUn6334.6;evm.TU.Cha06g008170;evm.TU.Cha13g001490;evm.TU.Cha10g001480;evm.TU.Cha15g008840;evm.TU.Cha14g000130;evm.TU.Cha15g008470;evm.TU.Cha04g002490;evm.TU.Cha14g012310;evm.TU.Cha01g014620;evm.TU.Cha11g000480;evm.TU.Cha14g001460;evm.TU.Cha11g000470;evm.TU.Cha15g000640;evm.TU.Cha08g008710;evm.TU.Cha06g004860;evm.TU.Cha13g008460;evm.TU.Cha02g002080;evm.TU.ChaUn7495.3;evm.TU.Cha13g004580;evm.TU.Cha06g019130;evm.TU.Cha06g004830;evm.TU.Cha03g021920;evm.TU.Cha06g004780;evm.TU.Cha09g011610;	25	**LysoPE 18:2; LysoPE 18:3; LysoPC 18:3; LysoPE 18:1;**	4
04075(Plant hormone signal transduction)	evm.TU.Cha01g018990;evm.TU.Cha09g013810;evm.TU.ChaUn5907.3;evm.TU.Cha03g001290;evm.TU.Cha03g011080;evm.TU.Cha09g014620;evm.TU.Cha11g006730;evm.TU.Cha02g018920;evm.TU.Cha09g012590;evm.TU.ChaUn12188.1;evm.TU.Cha01g011620;evm.TU.Cha01g025450;evm.TU.Cha	72	**Jasmonic acid;**	1

04g021510;evm.TU.Cha06g001380;evm.TU.Cha14
g010030;evm.TU.Cha06g001340;evm.TU.ChaUn1
3023.2;evm.TU.ChaUn7157.1;evm.TU.Cha10g004
610;evm.TU.Cha13g011850;evm.TU.Cha15g00306
0;evm.TU.Cha13g005780;evm.TU.Cha14g012470;
evm.TU.Cha01g008020;evm.TU.Cha02g018930;ev
m.TU.Cha06g001290;evm.TU.Cha06g001360;evm.
TU.Cha06g013790;evm.TU.Cha02g000180;evm.T
U.Cha09g014160;evm.TU.Cha03g006520;evm.TU.
ChaUn7487.1;evm.TU.ChaUn14186.2;evm.TU.Ch
a03g018010;evm.TU.Cha09g008360;evm.TU.Cha1
1g003400;evm.TU.Cha08g004350;evm.TU.Cha09g
001120;evm.TU.Cha14g000400;evm.TU.Cha11g01
0960;evm.TU.Cha13g007000;evm.TU.Cha05g0035
40;evm.TU.Cha01g022030;evm.TU.Cha06g004550
;evm.TU.ChaUn6460.1;evm.TU.Cha10g012500;ev
m.TU.Cha06g001320;evm.TU.Cha15g009870;evm.
TU.Cha01g016780;evm.TU.Cha11g010430;evm.T
U.Cha08g009600;evm.TU.ChaUn11907.2;evm.TU.
Cha02g018950;evm.TU.Cha06g001280;evm.TU.C
ha03g003950;evm.TU.Cha06g001350;evm.TU.Cha
08g014550;evm.TU.Cha12g002780;evm.TU.Cha01
g000980;evm.TU.ChaUn26553.1;evm.TU.ChaUn2
3146.1;evm.TU.ChaUn7586.1;evm.TU.Cha01g018
200;evm.TU.Cha13g006400;evm.TU.Cha15g01111
0;evm.TU.Cha05g002250;evm.TU.Cha07g009730;

evm.TU.Cha05g016100;evm.TU.ChaUn12349.3;evm.TU.Cha06g020330;evm.TU.Cha13g007060;evm.TU.Cha06g020210;	1

Table S6 Primers used in this study.

Name	Forward primer (5' – 3')	Reverse primer (5' – 3')
CcCp1	GCAAACCCAGGGCAACAT	ATGGCGAGGACGTTGATG
CcWDR83	TAATCGCTGGAGACGAGTTGA	TCTTGGGTTCATAGCCTTCG
neo	TCAAGCTGTTTGATGATTTCAGT	TTGGTGCGTTTGTCAAGCAA
hyg	GTCGTTTGACAAGATGGTTCA	CGTCTGCTGCTCCATACAA
Up-CcCp1	GgcggcctcgagGGATGTTGTTTGCCTGCG	CGGGGTACCTTTGGCGGTTGTGGGTGGA
Down-CcCp1	GCTCTAGAACCACCACACGACCTCGG	ACGCGTCGACGACCCACATGCAGCTCCGA
C-CcCp1	GAAGATCTATGCAGTTCTCCAACCTTGTC	GGACTAGTCCCAGACCGCAGGCGCTGA

Additional file 1. Statistically significantly differentially changed genes (SSTF) involved in lipid pathway.

Additional file 2. Statistically significantly differentially changed genes (SSTF) involved in plant hormone signal transduction.

Additional file 3. Statistically significantly differentially changed genes (SSTF) involved in flavonoid, flavonol and isoflavonoid biosynthesis pathway.

Additional file 4. Statistically significantly differentially changed genes (SSTF) involved in plant pathogen interaction.

Additional file 5. The significantly enriched metabolic pathways observed among all the differentially accumulated metabolites (DAMs). The comparison of DAMs in CCLJ vs CKLJ, CPmLJ vs CKLJ, CCZC vs CKZC and CPmZC vs CKZC were shown, respectively.

Additional file 6. Integrated transcriptome and metabolomic analysis indicate the differentially expressed genes and differentially accumulated metabolites upon *C. camelliae* CCA infection tea cultivars (Longjing 43, Zhongcha 108) compared with untreated (CK).

Additional file 7. Integrated transcriptome and metabolomic analysis indicate the differentially expressed genes and differentially accumulated metabolites upon *C. camelliae* Δ*CcCp1* infection tea cultivars (Longjing 43, Zhongcha 108) compared with untreated (CK).
